# Mathematical modelling and a systems science approach to describe the role of cytokines in the evolution of severe dengue

**DOI:** 10.1186/s12918-017-0415-3

**Published:** 2017-03-11

**Authors:** S. D. Pavithra Jayasundara, S. S. N. Perera, Gathsaurie Neelika Malavige, Saroj Jayasinghe

**Affiliations:** 1 Research and Development Centre for Mathematical Modelling, University of Colombo, Colombo, Sri Lanka; 2 Centre for Dengue Research, Faculty of Medicine, University of Sri Jayewardenepura, Nugegoda, Sri Lanka; 3 Department of Clinical Medicine, Faculty of Medicine, University of Colombo, Colombo, Sri Lanka

**Keywords:** Dengue, Fuzzy logic, Cytokines, Combined effect

## Abstract

**Background:**

Dengue causes considerable morbidity and mortality in Sri Lanka. Inflammatory mediators such as cytokines, contribute to its evolution from an asymptotic infection to severe forms of dengue. The majority of previous studies have analysed the association of individual cytokines with clinical disease severity. In contrast, we view evolution to Dengue Haemorrhagic Fever as the behaviour of a complex dynamic system. We therefore, analyse the combined effect of multiple cytokines that interact dynamically with each other in order to generate a mathematical model to predict occurrence of Dengue Haemorrhagic Fever. We expect this to have predictive value in detecting severe cases and improve outcomes. Platelet activating factor (PAF), Sphingosine 1- Phosphate (S1P), IL-1β, TNFα and IL-10 are used as the parameters for the model. Hierarchical clustering is used to detect factors that correlated with each other. Their interactions are mapped using Fuzzy Logic mechanisms with the combination of modified Hamacher and OWA operators. Trapezoidal membership functions are developed for each of the cytokine parameters and the degree of unfavourability to attain Dengue Haemorrhagic Fever is measured.

**Results:**

The accuracy of this model in predicting severity level of dengue is 71.43% at 96 h from the onset of illness, 85.00% at 108 h and 76.92% at 120 h. A region of ambiguity is detected in the model for the value range 0.36 to 0.51. Sensitivity analysis indicates that this is a robust mathematical model.

**Conclusions:**

The results show a robust mathematical model that explains the evolution from dengue to its serious forms in individual patients with high accuracy. However, this model would have to be further improved by including additional parameters and should be validated on other data sets.

**Electronic supplementary material:**

The online version of this article (doi:10.1186/s12918-017-0415-3) contains supplementary material, which is available to authorized users.

## Background

Dengue is a mosquito borne viral disease transmitted by female mosquitoes of the species Aedes aegypti and Aedes albopictus. In the recent decades there has been a dramatic increase of the dengue incidences around the world [1]. Each year around 500,000 people with severe dengue are hospitalized, with a large proportion being children. Out of those affected around 2.5% result in death [1]. Dengue has been a national concern in Sri Lanka with several outbreaks occurring and the incidence and severity of these epidemics keeps increasing [2]. Most infected person are asymptomatic, and develop dengue fever (DF), while a minority proceed to serious forms of dengue, dengue haemorrhagic fever (DHF) or dengue shock syndrome (DSS), which can be fatal [3]. A key mechanism of severity is leakage of fluid from blood vessels to surrounding tissues and the resultant drop in volumes within the vascular compartment and hypotension. This occurs for about 48 h and is referred to as critical phase [4]. At present there is no specific drugs against the illness. Therefore, early clinical diagnosis and careful body fluid management is critical to care of the severe ill [5]. In relation to early diagnosis, attempts have been made to identify early markers of dengue and cytokines that predict severity [6–8].

Increased vascular permeability is a main cause of DHF and cytokines, inflammatory lipid mediators and dengue NS1 antigen are thought to significantly contribute to this increase in vascular permeability [9–11]. Hence, several studies have attempted to identify the relationships between cytokines and dengue. In this study, we have attempted to use several cytokines and other inflammatory mediators to develop a mathematical model to predict the likelihood of developing DHF. For this model we have chosen three cytokines and two inflammatory lipid mediators due to their association with vascular leak in dengue and also with severe clinical disease. Sphingosine 1-phosphate (S1P), is a signalling lipid mediator and is considered to be important in maintaining endothelial barrier integrity [12]. It was shown that levels of S1P, were found to be low in DHF patients especially during the critical phase of acute dengue [13]. IL-1β was also found to associate with increase in vascular permeability and is thought to be predominantly released from platelets in patients with acute dengue [14]. IL-1β is shown to be released from dengue virus infected monocytes, which is thought to be due to the activation of the inflammasome [15, 16]. IL-10 levels have also shown to be higher in patients with DHF especially during secondary infections [17, 18]. In addition, it was recently shown that higher concentrations of NS1 antigen and serum IL-10 levels are associated with severe clinical disease in acute dengue infection [6, 19]. However, although IL-10 levels were found to be significantly higher in patients with DHF, it was not a good predictive marker when used alone due to the high variability [6]. Although TNF-α was initially found to be associated with DHF [20], more recent studies again has shown variable results [21, 22]. A main drawback of these studies is that they focus on the association of individual cytokines with clinical disease severity. However, when identifying markers of DHF it is important to take into consideration the dependencies and interaction of inflammatory mediators [23].

In recent times there has been an interest in the utility of a systems science approach that captures the combined and inter-related effects of multiple parameters in determining severity of illnesses [24]. Our study is an attempt to take a systems science view of severity and develop a mathematical model to capture the combined effect of multiple inflammatory mediators that are elevated in dengue. Therefore, in this study our objective is to develop a mathematical model that can detect patients proceeding to DHF level at an early stage by analysing the combined effect from the parameters sphingosine 1-phosphate (S1P), Interleukin- 1β (IL-1β), Tumor Necrosis Factor (TNF-α), Platelet Activating Factor (PAF) and Interleukin -10 (IL-10). It was recently shown that higher concentrations of NS1 antigen and serum IL-10 levels are associated with severe clinical disease in acute dengue infection [19]. The current study uses some of the published data and other sources to model the impact of multiple immune and other variables in predicting severity of dengue.

In our study a fuzzy logic based model is proposed to analyse the combined effect of inflammatory mediators to determine severity level of dengue. Fuzzy logic is now commonly used to model biological problems as it has the strength to handle imprecise information and uncertainties associated with decision making [25].

## Methods

### Preliminary analysis

The sample used for preliminary analysis and model validation consists of 11 adult patients with DF and 25 adult patients with DHF, recruited from the Colombo South Teaching Hospital, Sri Lanka. Model validation was supported through pre-existing data in [13] and [19]. The classification as to DF or DHF is performed according to 2011 WHO guidelines [1]. The patients in the sample are admitted at varying time points from onset of fever ranging from 72 to 144 h from onset of fever. Data are collected at several time points for a particular individual patient, each time point being 12 h apart. The number of times a patient is measured differs from individual to individual and hence there are missing values as not all time points are measured for all of the patients. Missing values are handled using multiple imputation.

**Fig. 1:**
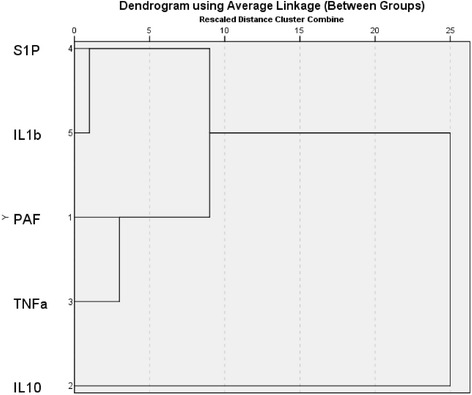
Dendrogram resulting in hierarchical clustering performed on the five cytokine and inflammatory mediators S1P, IL-1β, TNF-α, PAF and IL-10 on DHF patients at 96 h from onset of illness. SPSS statistical software is used to cluster the variables and squared Euclidian Distance is used as the distance measure. In the model development these parameters are combined with the Hamacher product and OWA operator according to this clustering output

Hierarchical clustering is performed on the parameter variables and the resulting dendrogram at 96 h from onset of fever is shown in Fig. 1. The clusters are formed at increasing level of dissimilarity and squared Euclidian Distance is used. SPSS statistical software is used to cluster the variables. It can be seen that S1P and IL-1β merges first, being the closest pair of clusters and TNF-α, IL-10 and PAF shows similar behaviour, resulting in two main clusters and the same two clusters could be seen for 96 and 120 h of onset of illness for DHF patients. These clustering output is used in deciding how to aggregate parameters with the Hamacher operator.

### Model development

Our model to determine dengue severity by analysing the combined effect of cytokines and inflammatory mediators is modelled using fuzzy logic concepts. With the ambiguity and vagueness associated with decision making in dengue and medicine in general, fuzzy logic is commonly used in modelling these phenomena as it has the ability to explain the uncertainties associated with these complex systems. Fuzzy models have the ability to handle these imprecise components and perform with high accuracy as fuzzy models are robust to variation in symptom parameters [26]. Dengue pathogenesis is complex and still not fully understood [27]. Disease severity can depend on dengue serotype [28] and it is believed that antibody dependent enhancement can also play a role in determining severity [29]. Disease diagnosis itself include uncertainties as symptoms could vary from patient to patient, similar symptoms can be common to various diseases and human reasoning itself is imprecise [25]. Therefore, strict rules as in classical logic is not suitable to handle biological problems that involve inherent uncertainty. Also, fully stochastic models cannot be adopted as the underlying probability distributions are unknown [30]. Fuzzy logic provides a platform to interpret vague human descriptions in natural linguistic terms and can successfully handle imprecision and uncertainty and is a useful modelling tool especially under limited data [31].

Study in [32] has used fuzzy expert system to detect asthma and chronic obstructive pulmonary disease. Using parameters such as fever, nocturnal symptoms, oral steroids etc. the model produced a scale of 1-10 to measure the severity level of asthma, tuberculosis and chronic obstructive pulmonary disease. Fuzzy expert system for diabetes has also been developed [33]. In this system triangular membership functions with Mamdani Inference was used and it achieved an accuracy of 85.03%, which was higher than previously developed methods to detect diabetes. Two approaches based on Artificial Neural Networks (ANN) and Adaptive Neuro-Fuzzy Inference System (ANFIS) were used for identifying heart disease from a large data set on patients in [34]. In another study, the fuzzy system for detecting heart disease had a 94% accuracy to that of a medical expert’s decision [35]. Similar fuzzy expert systems to diagnose liver disorders are also available [36]. This expert system used triangular and trapezoidal membership functions and the achieved accuracy was 91%. Fuzzy IF-Then rule based study was carried out for the diagnosis of the haemorrhage and brain tumour disease to determine the probability of disease [37]. All these fuzzy expert systems are rule based and MATLAB fuzzy logic tool box was used, but in our model we didn’t use a rule based approach, rather we used fuzzy intersection operator, Hamacher product and the Ordered Weighted Aggregation (OWA) operator.

#### Approach to model development

In classical logic every statement is either true or false. But, in medical diagnosis it is not possible to make decisions based on these crisp distinctions. In fuzzy logic this strict convention is reduced and allows partial membership in a set. The degree to which a particular member belongs to a set is denoted by the degree of fuzziness and this is mapped through a fuzzy membership function [38]. Each element of a fuzzy set is mapped into a real number in the interval [0, 1].

The row values of cytokine and inflammatory mediator values are ‘fuzzified’ through their respective membership functions. As the objective of this study is to consider the combined effect from the parameters, Hamacher and OWA operators are selected as suitable fuzzy operators to combine parameters. Impact from the important variables to the model is intensified through fuzzy ‘concentration’. The model outputs a final value which measures the unfavourability to attain severe dengue. Based on this index value it can be decided whether this patient is a potential DF or a DHF patient.

For technical details of fuzzy set, membership functions, Hamacher operator and concentration see Additional file 1 (A1).

#### Membership function development

The five inflammatory mediators S1P, IL-1β, TNF-α, PAF and IL-10 are analysed in combination. When developing membership functions, knowledge acquisition from interviews with medical experts is a common practice [31, 39, 40]. Furthermore, previous studies that are carried out to determine the influence from these individual cytokines on dengue disease severity are also used to determine membership values. This enabled to develop our model independently of sample data. Since the rate of change of cytokines is not significant over time, trapezoidal membership functions are used to ‘fuzzify’ the input parameter values. Trapezoidal membership functions are commonly used to model problems in biology because of its easy construction and interpretation [35, 36]. In our model the membership functions measure how unlikely it is to develop DHF.

Above 50% of patients with DHF have shown S1P levels below 0.5 μM at some time point in their illness and only 10% of DF patients show S1P levels below 0.5 μM. For the membership function for S1P the cut off value for DHF patients is chosen as 0.5 μM. Also it shows that when compared with DF patients, DHF patients have significantly lower S1P levels throughout the course of illness [13]. The membership function for S1P is, *μ*_*S*_(*x*),

1 $$ {\mu}_S(x)=\left\{\begin{array}{c}\hfill 0\kern2em ; x\le 0.5\hfill \\ {}\hfill x-0.5\kern2em ;0.5< x<1.5\hfill \\ {}\hfill 1\kern1.75em ; x\ge 1.5\hfill \end{array}\right. $$

DF patients have shown to have an IL-1β level ranging from 0 to 33.7 pg/ml with a median of 30.5 pg/ml and DHF patients an IL-1β range of 0–62.3 pg/ml with a median of 33.5 pg/ml [41]. Therefore, the membership function for IL-1β is, *μ*_*β*_(*x*),

2 $$ {\mu}_{\beta}(x)=\left\{\begin{array}{c}\hfill 1\kern1.25em ; x\le 30.5\hfill \\ {}\hfill \frac{33.5- x}{3}\kern1.25em ;30.5< x<33.5\hfill \\ {}\hfill 0\kern1.5em ; x\ge 33.5\hfill \end{array}\right. $$

IL-10 concentration of DHF patients has shown a median 110.8, SD ± 27.1 pg/ml and DF patients a median of 15.5, SD ± 5.3 pg/ml. IL-10 levels are significantly elevated in DHF patients than in DF patients [42]. The membership function for IL-10 is, *μ*_10_(*x*),

3 $$ {\mu}_{10}(x)=\left\{\begin{array}{c}\hfill 1\kern1.25em ; x\le 20\hfill \\ {}\hfill \frac{110- x}{90}\kern1.25em ;20< x<110\hfill \\ {}\hfill 0\kern1.5em ; x\ge 110\hfill \end{array}\right. $$

The mean value for TNF-α for DF patients is indicated as 14.10, SD ± 24.0 pg/ml and for DHF patients mean 29.95, SD ± 39.5 pg/ml. TNF-α is higher in DHF and shock patients than in DF patients [43]. In the model the membership function for TNF-α is, *μ*_*α*_(*x*),

4 $$ {\mu}_{\alpha}(x)=\left\{\begin{array}{c}\hfill 1\kern1.25em ; x\le 15\hfill \\ {}\hfill \frac{30- x}{15}\kern1.25em ;15< x<30\hfill \\ {}\hfill 0\kern1.5em ; x\ge 30\hfill \end{array}\right. $$

PAF levels are found to be significantly higher in DHF patients [10]. Also in that study 72% of DF patients never showed a rise of PAF level above 100 ng/ml and median PAF level for DHF patients is 335.2 ng/ml while DF patients indicate to have a median value of 47.63 ng/ml. Therefore, the membership function for PAF in the model is, *μ*_*P*_(*x*),

5 $$ {\mu}_P(x)=\left\{\begin{array}{c}\hfill 1\kern1.25em ; x\le 10\hfill \\ {}\hfill \frac{100- x}{90}\kern1.25em ;10< x<100\hfill \\ {}\hfill 0\kern1.5em ; x\ge 100\hfill \end{array}\right. $$

**Fig. 2:**
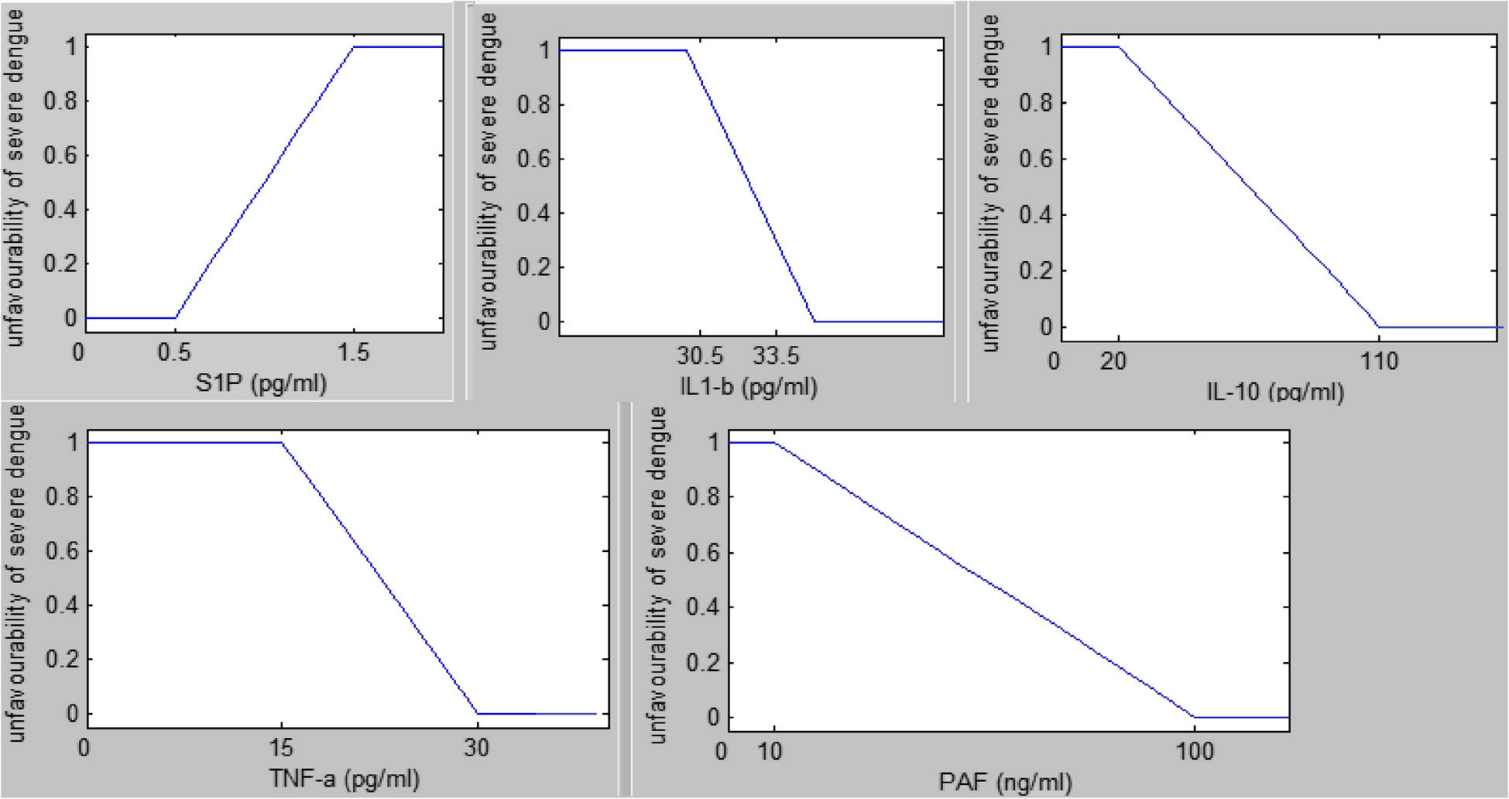
Model membership functions for S1P, IL-1β, IL-10 TNF-α and PAF

The trapezoidal-shaped membership functions are illustrated in Fig. 2.

#### Choice of fuzzy operator

Since the overall combined effect from S1P, IL-1β, TNF-α, IL-10 and PAF is considered to determine the severity level, the proposed operator must satisfy certain properties [44]. Let *Y, Z* be two cytokine parameters and *U*_*Y*_(*x*), *U*_*Z*_(*x*) measure their respective degree of unfavourability to attain DHF. Then,

- If *Y* is favourable to DHF and *Z* is favourable to DHF, then *U*_*Y* ∩ *Z*_(*x*) < min(*U*_*Y*_(*x*), *U*_*Z*_(*x*)).
- If *U*_*Y*_(*x*) < *U*_*Z*_(*x*) < 1, then the effect that a decrease of *U*_*Y*_(*x*) has on *U*_*Y* ∩ *Z*_(*x*) may depend on *U*_*Z*_(*x*).
- If *U*_*Y*_(*x*) and *U*_*Z*_(*x*) < 1, then the effect that an increase of favourability level of *Y* has on *U*_*Y* ∩ *Z*_(*x*) can be erased by a decrease of favourability of *Z*.

Since the Hamacher product as defined in A1-(1) possesses these three properties it is used in our model to combine the effect from cytokine parameters. Hamacher operator has been previously used successfully to combine the effect that rain fall and temperature can have on dengue disease transmission [30].

#### Development of the model

With clustering results as shown in Fig. 1 we are able to divide the five parameters mainly into two groups; one with S1P and IL-1β and the other with TNF-α, IL-10 and PAF. Therefore, the Hamacher product is separately used on the two main cytokine groups as shown in Eqs. (6), (7) and (8).

In the model the three parameters TNF-α, PAF and IL-10 are subjected to ‘concentration’ as previous studies clearly indicate that these parameters are significantly elevated in DHF patients than in DF patients [10, 17, 43]. Therefore, in order to amplify the effect from these cytokines and to allocate them a higher weight in the model, the membership values of TNF-α, PAF and IL-10 are concentrated.

The Hamacher product between S1P and IL-1β is

6 $$ \mathrm{H}1=\frac{\mu_S(x)\ast {\mu}_{\beta}(x)}{\mu_S(x)+{\mu}_{\beta}(x)-{\mu}_S(x)\ast {\mu}_{\beta}(x)} $$

where *μ*_*S*_(*x*), *μ*_*β*_(*x*) are the membership values of S1P and IL-1β obtained from (1) and (2) respectively.

The Hamacher product between TNF-α and IL-10 is

7 $$ \mathrm{H}=\frac{\mu_{\alpha}{(x)}^{\gamma}*{\mu}_{10}{(x)}^{\upvarphi}}{\mu_{\alpha}{(x)}^{\upgamma}+{\mu}_{10}{(x)}^{\upvarphi}-{\mu}_{\alpha}{(x)}^{\upgamma}*{\mu}_{10}{(x)}^{\upvarphi}}\kern5em \mathrm{where}\kern0.5em \upgamma, \upvarphi >1 $$

Where *μ*_10_(*x*) , *μ*_*α*_(*x*) are the membership values of TNF-α and IL-10 obtained from (3) and (4) respectively. Here TNF-α and IL-10 are concentrated by γ amount.

The Hamacher product between TNF-α, IL-10 and PAF is

8 $$ \mathrm{H}2=\frac{\mathrm{H}\ast {\mu}_p{(x)}^{\updelta}}{\mathrm{H}+{\mu}_p{(x)}^{\updelta}-\mathrm{H}\ast {\mu}_p{(x)}^{\updelta}}\kern8em \mathrm{where}\kern0.5em \updelta >1 $$

where *μ*_*p*_(*x*) is the membership values of PAF obtained from (5) and H is obtained in (7). Here PAF values are concentrated by δ amount. The Hamacher operator value resulting from S1P, IL-1β (6) and the Hamacher operator value resulting from TNF-α, IL-10 and PAF (8) is combined through the OWA operator defined in A1-(2).

So the OWA operator used in the model is

9 $$ \mathrm{O}\mathrm{W}\mathrm{A}=\uplambda \ast \mathrm{MAXIMUM}\left(\mathrm{H}1,\mathrm{H}2\right)+\left(1\hbox{-} \uplambda \right)\ast \mathrm{MINIMUM}\left(\mathrm{H}1,\mathrm{H}2\right) $$

where λ is OWA weight defined in A1-(3).

#### Model parameters

In the model the parameters TNF-α, PAF and IL-10 are concentrated by 1.1, 1.2 and 1.1 respectively. Accordingly, the model parameter values of γ, δ and φ are 1.1, 1.2 and 1.1 respectively. PAF is concentrated more than the other two as it plays a highly significant role in determining severity. Concentration is limited to a small amount as otherwise it would affect the operator values of DF patients.

The optimal λ value is determined by analysing the accuracy of the model for various values of λ. Table 1 summarizes these results. λ = 0.2 and λ = 0.3 make the DF operator values too biased towards DHF patients. In fact, when λ = 0.2 at 120 h from onset of illness 7 out of 8 DF patients are misclassified. When λ = 0.4, model performs well when all three time points are considered and it is better in classifying DF patients. Therefore, optimal λ is chosen as 0.4. Thus, from A1- (3) and A1-(4) by letting *l* = 0.3 and *m* = 0.8 the OWA weights chosen for the model are 0.4 and 0.6.

**Table 1:** λ values and model performance

λ	Accuracy at 96 h from onset of illness	Accuracy at 108 h from onset of illness	Accuracy at 120 h from onset of illness
0.2	76.19%	85.00%	62.96%
0.3	71.4%	85.00%	62.96%
0.4	71.4%	85.00%	76.92%

The ‘orness measure’ of the model as calculated according to A1-(5) is 0.4. This means that the OWA operator does not work entirely as an AND operator and to some degree (orness measure of 0.4) it works as an OR operator. Hamacher product acts as an AND operator, thus it further reduces the operator values making the model to be too biased towards DHF patients. But, by using OWA operator to aggregate the Hamacher product of S1P, IL-1β and the Hamacher product of TNF-α, IL-10 and PAF it allows to compensate the over intensification caused from Hamacher product to a certain extent and provide a better way to distinguish DF and DHF patients.

#### Construction of ambiguous region

**Fig. 3:**
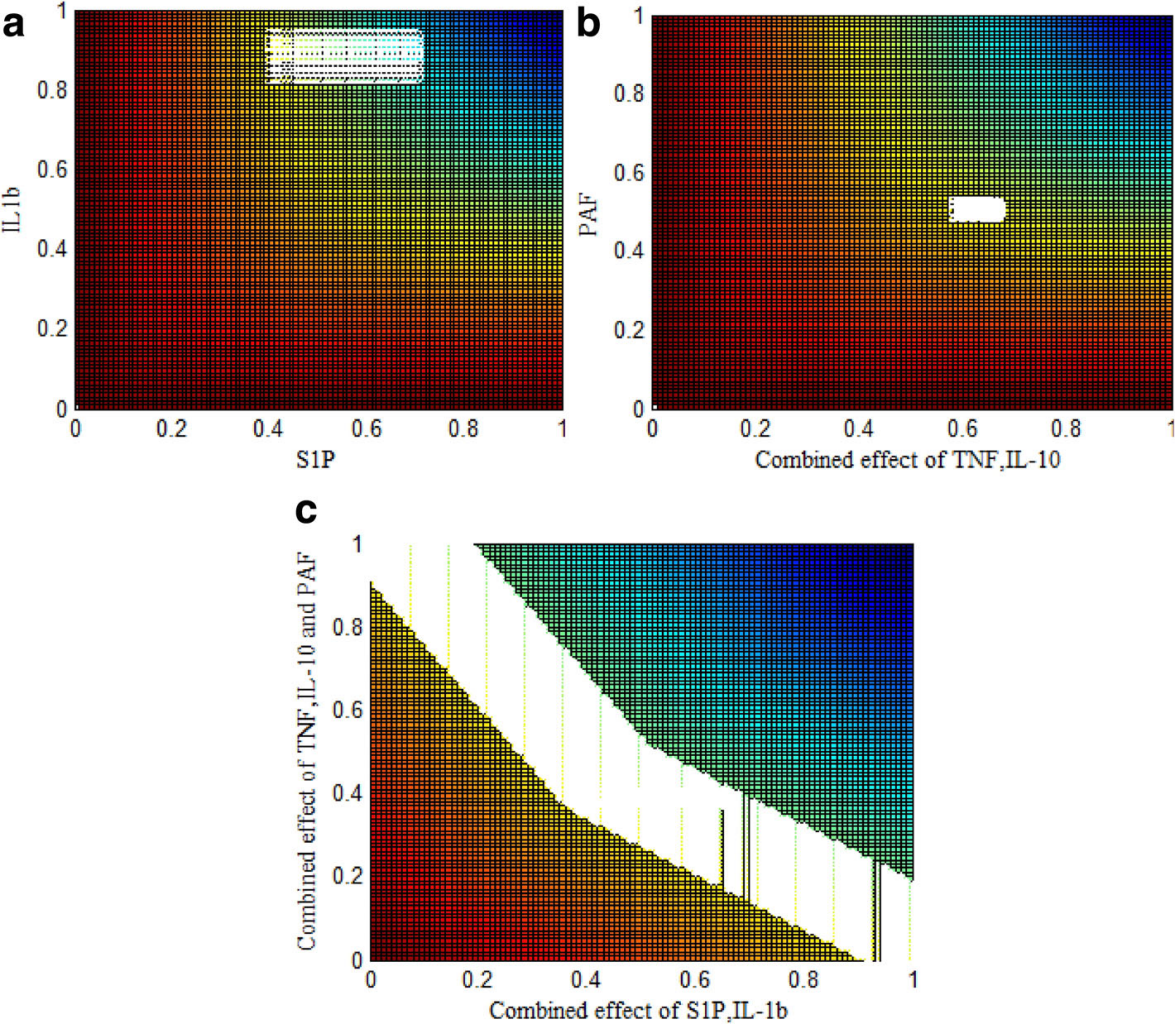
Ambiguous region for first cluster of cytokines (S1P, IL-1β) (**a**), second cluster of cytokines (IL-10, TNF-α, PAF) (**b**), final ambiguous region in model (**c**). Ambiguous region is indicated by the *white colour* region. This is the region in where it is not possible to make a precise decision as to whether the patient is severe or non-severe based on the model value

The overall ambiguous region is determined by using ambiguous regions of individual cytokines. In this region it cannot be determined specifically whether the patient is DHF or a DF patient. The ambiguous levels of individual cytokines are determined using the cytokine values which result in around 0.5 degree of membership values. As the membership functions are developed independently of sample data, the resulting overall ambiguous region also becomes independent of sample data. The individual ambiguous levels that are used for each parameter are 0.9–1.2 pg/ml for S1P, 30.7–31 pg/ml for IL-1β, 17.5–19 pg/ml for TNF-α, 38–40.5 pg/ml for IL-10 and 48–50 ng/ml for PAF. Separate ambiguous levels of the cluster S1P,IL-1β and the cluster from IL-10,PAF,TNF-α is given in Fig. 3(a) and (b) respectively and the final ambiguous region of the model is displayed in Fig. 3(c).

#### Algorithm of the fuzzy decision support system

- INPUT - Input the fuzzy set for S1P, IL-1β, TNF-α, PAF and IL-10
- OUTPUT - Operator value which measures unfavourability to attain DHF.

PROCEDURE

1. Input the crisp values (raw patient data) on cytokines S1P, IL-1β, TNF-α, PAF and IL-10.
2. Generate the fuzzy membership values for each cytokine using respective membership functions.
3. Concentrate the membership values of TNF-α, PAF and IL-10 by 1.1, 1.2 and 1.1 respectively.
4. Obtain Hamacher product (H1) of the variables S1P and IL-1β,
  $$ \mathrm{H}1=\frac{\mu_S(x)\ast {\mu}_{\beta}(x)}{\mu_S(x)+{\mu}_{\beta}(x)-{\mu}_S(x)\ast {\mu}_{\beta}(x)} $$
  where *μ*_*S*_(*x*), *μ*_*β*_(*x*) are the membership values of S1P and IL-1β respectively.

5: Obtain Hamacher product (H) of the variables TNF-α and IL-10
  $$ \mathrm{H}=\frac{\mu_{\alpha}{(x)}^{1.1}\ast {\mu}_{10}{(x)}^{1.1}}{\mu_{\alpha}{(x)}^{1.1}+{\mu}_{10}{(x)}^{1.1}-{\mu}_{\alpha}{(x)}^{1.1}\ast {\mu}_{10}{(x)}^{1.1}} $$
  where *μ*_*α*_(*x*), *μ*_10_(*x*) are the membership values of TNF-α and IL-10 respectively.

6: Obtain Hamacher product (H2) of the variable PAF and H
  $$ \mathrm{H}2=\frac{\mathrm{H}\ast {\mu}_p{(x)}^{1.2}}{\mathrm{H}+{\mu}_p{(x)}^{1.2}-\mathrm{H}\ast {\mu}_p{(x)}^{1.2}} $$
  where *μ*_*p*_(*x*) is the membership values of PAF and H is obtained in step 5.

7: Obtain the OWA operator of H1 and H2 with weights 0.4 and 0.6

8: Output final operator value measuring unfavourability to attain DHF.

END

MATLAB codes are provided in Additional File 2.

## Results

### Model operator regions

Three main regions are identified in the model; non severe (DF), severe (DHF) and ambiguous region. If the model output value is below 0.36 the patient is considered as DHF and if the model output value is above 0.51 the patient is considered as DF. A region of ambiguity is detected in the model for the value range 0.36 to 0.51. In this region it cannot be determined specifically whether the patient is DHF or a DF patient.

### Model validation

**Fig. 4:**
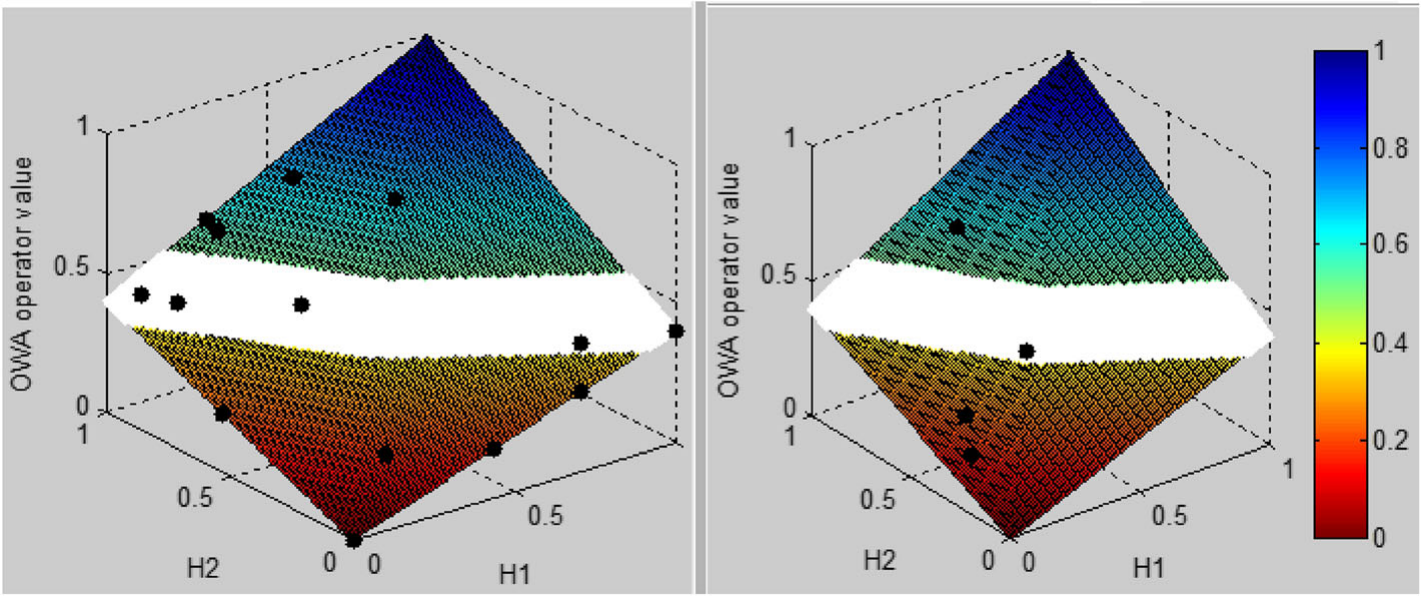
Model validation results for DHF (*left*) and DF (*right*) patients at 96 h from onset of fever. H1 refers to the Hamacher result of S1P and IL-1β and H2 refers to the Hamacher result of TNF-α, PAF and IL-10. In the scale, values closer to 1 (*blue shaded area*) represent non severe (DF) region and the scale values closer to 0 (*red shaded area*) represent severe region (DHF). The ambiguous region in where it is difficult to make a precise decision as to whether the patient is heading towards severe or non-severe region is indicated in the *white* region. The *black dots* represent the model output result of each patient and the severity level can be determined depending on the region the patient falls into. For the figure on left (DHF) we expect more patients to fall in the *red* shaded area while for the figure on right (DF) we expect more patients to fall in the *blue* shaded area

**Fig. 5:**
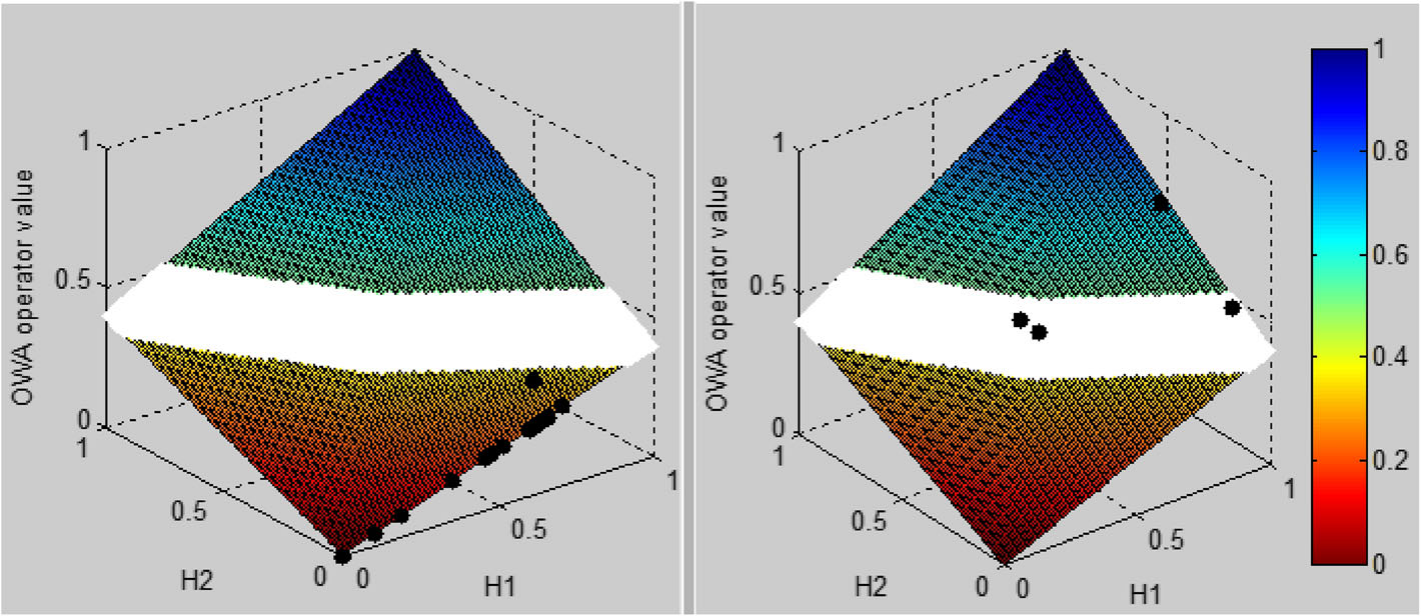
Model validation results for DHF (*left*) and DF (*right*) patients at 108 h from onset of fever. H1 refers to the Hamacher result of S1P and IL-1β and H2 refers to the Hamacher result of TNF-α, PAF and IL-10. The interpretation of the colours, regions and dots of this figure are as same as that is given in Fig. 4

**Fig. 6:**
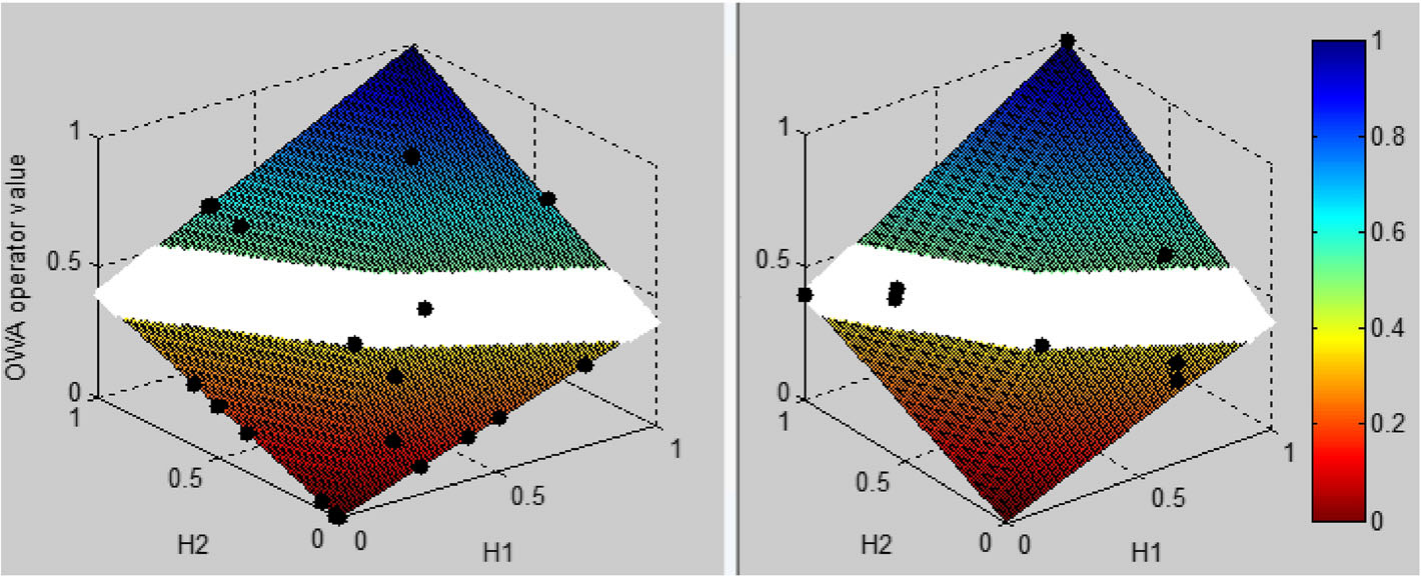
Model validation results for DHF (*left*) and DF (*right*) patients at 120 h from onset of fever. H1 refers to the Hamacher result of S1P and IL-1β and H2 refers to the Hamacher result of TNF-α, PAF and IL-10. The interpretation of the colours, regions and dots of this figure are as same as that is given in Fig. 4

The model is validated at 96, 108 and 120 h from onset of fever using DF and DHF patients in the sample as shown in Figs. 4, 5 and 6 respectively. The model’s validation at these time points is justified as the critical phase occurs often after the third day of fever, usually around the fifth or sixth day of illness with defervescence [45]. The model is validated using sample data collected from Colombo South Teaching Hospital. The sample included data for S1P, IL-1β, TNF-α, IL-10 and PAF collected at various time points from onset of illness. The data collected from patients were given as input to the model, which would then output a value that measure the disease severity level. This was done separately for 96, 108 and 120 h from onset of fever. Depending on this model output value it can be determined whether the patient is DF, DHF or in the ambiguous region. In the sample data set the medical experts had made a final diagnosis of the patient and have made the classification as to whether the patient is DF or DHF based on the 2011 WHO guidelines [1]. So, as to assess the validity of our developed model we compared the model output result with the medical expert’s result. Then if the model decision and the medical expert’s decision are the same it was considered as a correct output from the model and if the two decisions differ, it was considered as an incorrect decision from the model (misclassification).

Using these validation results the accuracy of the model is determined. The accuracy of the model is calculated as

$$ \mathrm{Accuracy}=\frac{\mathrm{correctly}\ \mathrm{classified}\ \mathrm{DHF} + \mathrm{correctly}\ \mathrm{classified}\ \mathrm{DF}\ }{\mathrm{Total}\ \mathrm{DHF} + \mathrm{Total}\ \mathrm{DF}}. $$

When determining the accuracy the patients that fall in the ambiguous region are not considered as misclassified as ambiguity does not suggest an incorrect classification. The model’s accuracy at 96,108 and 120 h from onset of fever is displayed in Table 2.

**Table 2:** Model accuracy for 96, 108 and 120 h from onset of fever

Time from onset of fever	Model accuracy
96 h	71.43%
108 h	85.00%
120 h	76.92%

Both DHF and DF patients in the test sample are validated using the model. Table 3 illustrates the distribution of DF and DHF patients according to the region which they are categorized by the model. At 120 h from onset of fever there are four DHF patients (21.05%) with model operator values above 0.6. However, when the model operator values over time for these patients is observed, it can be seen that at time points before 120 h the model has indeed identified them as DHF patients, revealing the model’s capability to perform as an early predictive marker.

**Table 3:** Distribution of DF and DHF patients in severe, ambiguous and non-severe regions at 96, 108 and 120 h from onset of fever

Time from onset of fever	Patient severity level	Percentage in severe region	Percentage in ambiguous region	Percentage in non-severe region
96 h	DHF	29.4%	47.1%	43.5%
	DF	50%	25%	25%
108 h	DHF	100%	0%	0%
	DF	0%	75%	25%
120 h	DHF	73.68%	5.26%	21.05%
	DF	28.57%	42.85%	28.57%

Also, as seen from Table 3, high percentage of DF patients has fallen into severe and ambiguous regions. Furthermore, in four instances, patients with DF have shown operator values below 0.36. This indicates that the model is slightly biased at moving patients to severe region.

**Fig. 7:**
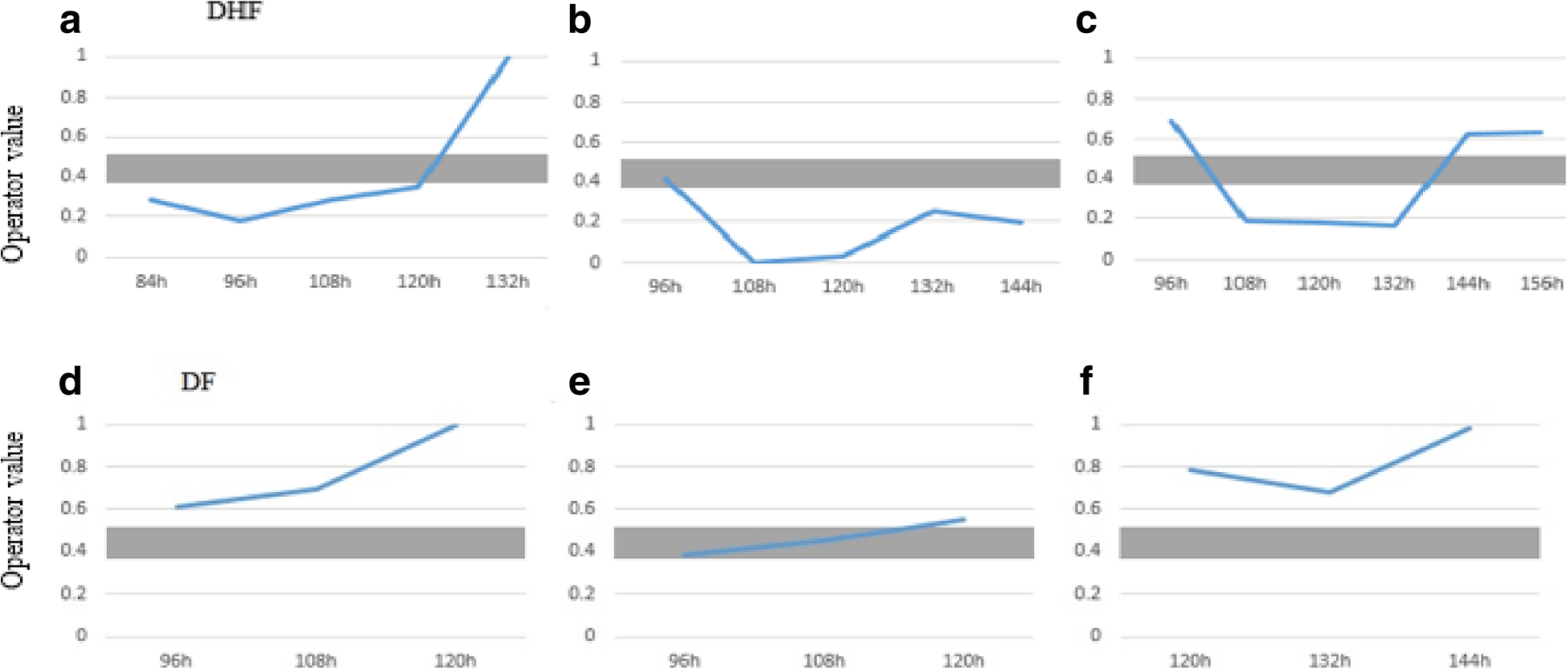
Change of model values over time for 3 DHF patients (**a**, **b**, **c**) and 3 DF (**d**, **e**, **f**) patients. Ambiguous region is shaded. Region above the shaded area is non-severe region and the area below is severe region

The model’s behaviour as it changes over time is analysed for individual patients. Fig. 7 shows this behaviour for three DHF and three DF patients. On admission most DHF patients as seen in Fig. 7 (b) and (c) show an operator value in the ambiguous region or in non-severe region, but as time progresses they move onto severe region and remains in this region for some time. However as shown in Fig. 7 (a) and (c) as they reach their final time point, they indeed move onto non-severe region. Therefore, from Fig. 7 it can be seen that the model indeed follows the expected clinical behaviour of DHF patients.

### Sensitivity analysis

**Fig. 8:**
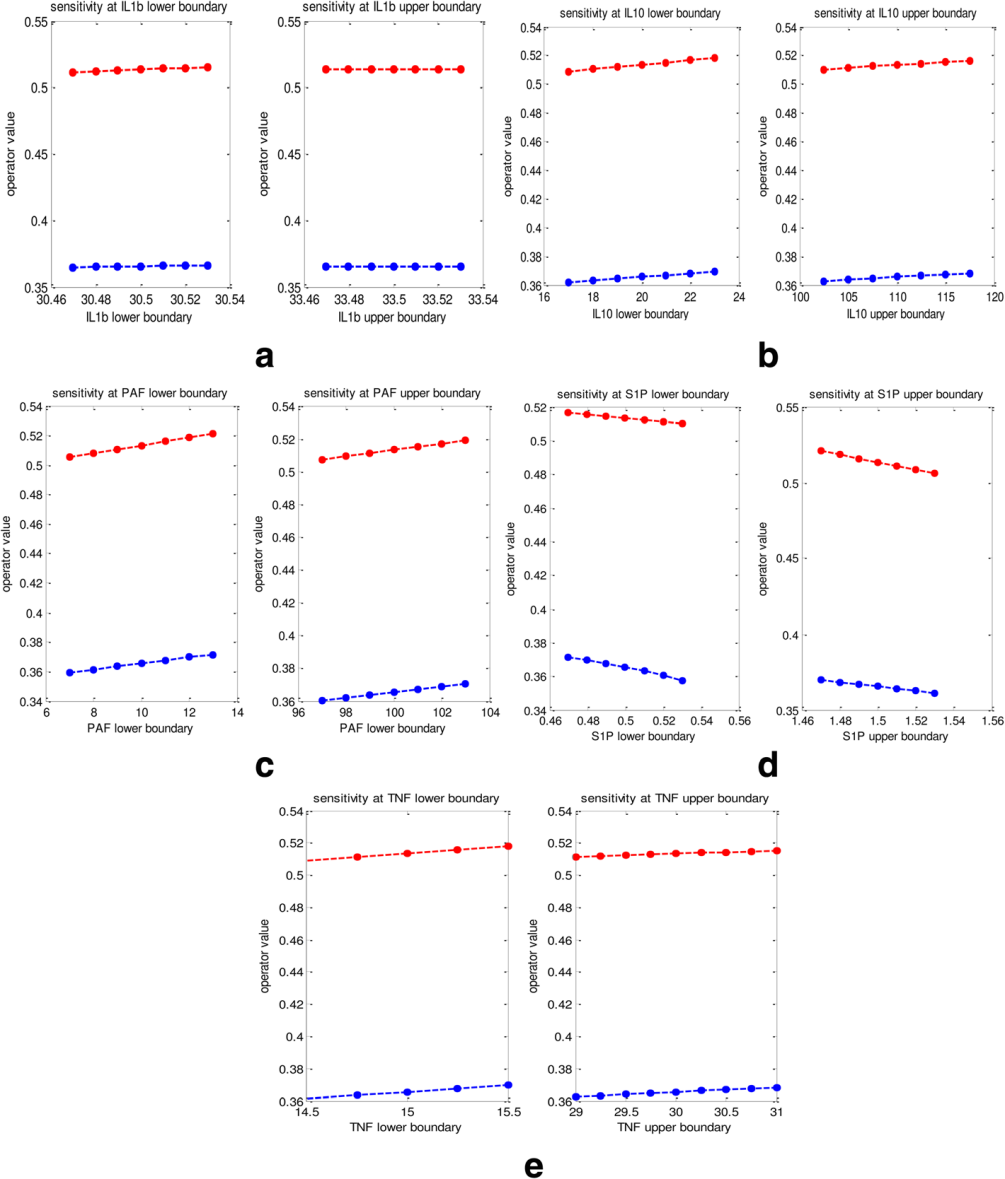
Change of lower and upper boundary values of the ambiguous region when the cut off (boundary) values of the membership functions are changed within a small range of the cytokines IL-1β (**a**), IL-10 (**b**), PAF (**c**), S1P (**d**), TNF- α (**e**). The *blue line* represents the lower level of the ambiguous region and the *red line* represents the upper level of the ambiguous region. Behaviour of the ambiguous region for a change in the lower cut off value of the membership function is displayed in the left of figure and the behaviour of the ambiguous region for a change in the upper cut off value of the membership function is displayed in the right of figure of each (**a**), (**b**), (**c**), (**d**) and (**e**)

Sensitivity analysis is performed in order to determine how the model operator values and the categorization of patients would change when the degrees of fuzziness are changed. In Fig. 8 the boundary values of each of the membership functions are changed by a small amount and the behaviour of the lower and upper limit of the ambiguous regions are analysed. The existing ambiguous region has a lower limit of 0.36 and upper limit of 0.51. As it can be seen from Fig. 8 as the boundary values of each of the membership functions are changed by a small amount the lower and upper limit of ambiguous regions do not change rapidly indicating a robust model.

**Fig. 9:**
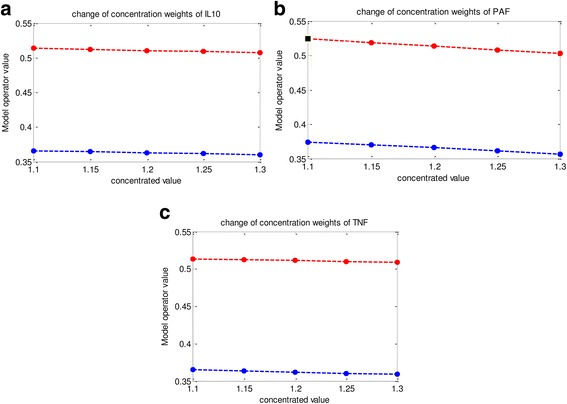
Behaviour of ambiguous region for a change in concentration weights for IL-10 (**a**), PAF (**b**), TNF-α (**c**). The *blue line* represents the lower level of the ambiguous region and the *red line* represents the upper level of the ambiguous region

In the model the parameters TNF-α, PAF and IL-10 are concentrated by 1.1, 1.2 and 1.1 respectively. A sensitivity analysis is performed on the weights on which the parameters are concentrated in order to determine how the ambiguous regions would change accordingly. As it can be seen from Fig. 9 as the concentration weights of the three parameters are changed by a small amount the lower and upper limit of ambiguous regions do not change rapidly indicating a robust model.

From Figs. 8 and 9 it can be concluded that this is a robust model and the classification of patients as to whether DF or DHF would not change when the model parameters are subjected to a small change from their existing values.

## Discussion

The model developed to predict the dengue severity performs well with considerable accuracy at all time points with the highest accuracy of 85.00% being achieved at 108 h from onset of fever. At 108 h from onset of fever, none of the DHF patients are misclassified or have fallen into the ambiguous region. This is important as at this time point the model does not succumb to the more serious error of misclassifying DHF patients. However, model performance at 96 h from onset of fever needs to be further improved as early detection would help clinicians to institute appropriate treatment before the patient enters the critical phase of infection [45]. At 96 h from onset of illness, 43.5% of DHF patients are classified as non-severe, though they are correctly classified at the next time point of 108 h. This discrepancy is likely to be due to the cytokine changes not being maximal at 96 h.

Also, in the model DF patients tend to get classified as either ambiguous or severe. Although our approach eliminates the possibility of classifying severe patients as non-severe, this is not ideal as when non severe patients are classified as severe we would not be able to meet up with the optimal resource allocation. The model is biased towards DHF detection because of the use of Hamacher product. The Hamacher product with the intersection operation, is able to intensify the risk level when the combined effect of cytokines is considered. To reduce this over intensification to a certain extent and to provide a better way to distinguish between DF and DHF patients the OWA operator is used as it compensates the over intensification caused due to the use of Hamacher product as it works with an ‘orness measure’.

Majority of the previous studies that have been conducted to analyse the association of cytokines and inflammatory mediators on dengue severity have focused on analysing the effect with respect to individual cytokines [6, 10, 13, 17, 18, 43]. However, as it was discussed in the introduction section, it is of importance to consider the combined effect from cytokines as the interactions, inter dependencies and compensations between parameters can have an impact in determining disease severity than when it is analysed individually [23]. As, the Hamacher product possess these properties it was used in our study to consider the cumulative effect from these parameters [44].

Several effective models have been developed based on fuzzy rules for the detection of dengue severity level. In the model in [46] the Mamdani fuzzy inference system is based on physical symptoms and laboratory reports as inputs. The clinical symptoms include fever, gastro intestinal symptoms, headache, body aches, skin rash, and retro-orbital pain. The system gives the output as “no dengue”, “probable dengue” and “confirmed dengue”. In the mobile application for dengue detection using fuzzy logic, the inputs are fever, skin rash, spontaneous haemorrhaging and tourniquet test [39]. These symptom-based models are useful and work with accuracy, especially in a field setting. However, these symptoms are not specific to dengue (e.g. other diseases such as chikungunya too have very similar symptoms) making it difficult to determine in the presence of co-existing epidemics [5]. In contrast, our model is based on cytokines and is more applicable in a health care setting where blood sampling is available. It is based on our understanding of the pathogenesis. Severe dengue affects the function of endothelial cells and inflammatory mediators are known to play a role in dengue disease severity [6, 7, 9, 10, 13, 18]. Therefore, our model is more objective as it relies on the measurements of the blood, rather than on symptoms. To our knowledge this is the first attempt at developing a fuzzy logic based decision system for dengue severity prediction based on combined interaction of cytokines and inflammatory mediators.

An ANFIS approach is used in [47] to construct diagnostic models using symptoms of dengue patients. In this study, initially an ANFIS model is developed and then it is further improved by using clustering algorithm. This model achieved an accuracy of 86.13%. ANFIS uses properties of ANN in developing fuzzy membership functions. An ANN approach in [48] classified the risk of dengue patients with an accuracy of 96.27%. To use ANN and ANFIS techniques it requires a larger data set as the data set has to be divided as training data set and testing data set and the model is trained using this sample training data set. The models based on these methods have acquired higher accuracy than our model. However, with the limited data set that we have, (at 96 h from onset of fever number of DHF patients 17, DF patients 4. At 108 h from onset of fever number of DHF patients 16, DF patients 4 and at 120 h from onset of fever number of DHF patients 19, DF patients 8) ANN or ANFIS method is not feasible. As we could not afford to use the sample data to develop the model, the model membership values were determined through previous studies [10, 13, 41–43] thus, making it being independent of sample data. This gave us the opportunity to fully utilize the limited data set for model validation. Also, with our approach no overfitting error occurs as the model is independent of data.

Boruta algorithm, which works well on significantly larger data sets was used in [23] to incorporate the effect of interdependency between cytokines. A classification and regression tree (CART) analysis performed on a cohort of Thai children analysed at 72 h from onset of illness achieved a 97% sensitivity in detecting patients who proceeded into DSS [49]. This decision tree algorithm used white blood cell count, percent monocytes, platelet count and haematocrit to make decisions. CART decision tree based on clinical and laboratory parameters including platelets, IL-10 and lymphocyte resulted in a model with an accuracy of 84.6% for DHF and 84.0% for DF and identified IL-10 and platelet counts as the most informative parameters [50]. Even with limited data with the fuzzy approach that we took, we were able to achieve an overall accuracy of 85.00% at 108 h from onset of fever. If much larger data set was present we could have adopted an approach of decision tree or ANN as a better selective approach on how the variables could be combined with the Hamacher and OWA operator and can look into improving the accuracy of our model. However, the limited small data set that we have, restricted us from using these machine learning techniques.

As we are working with a small sample size and in order to generalize our model performance we compared our model with previously developed models that are based on different techniques and also, performed a sensitivity analysis. Sensitivity analysis is highly important when working with a small sample size as, in these limited sample sizes, a small change in the patient decision can hugely affect the overall model performance. However, from Figs. 8 and 9, it can be seen that for a small change in the cut off values of the membership functions and the concentration levels the patient categorization remains unchanged. Therefore, even though we are working with a small sample size, sensitivity results indicate that the model is robust to change.

Although this mathematical model performs with high accuracy and is robust there are certain limitations and further improvements that can be incorporated to the model. As previous studies have shown that S1P levels are significantly correlated with platelet counts in DHF patients [10] and IL-10 levels are significantly and inversely correlated with lymphocyte counts [6], the performance of the model when cytokines are modelled with other clinical parameters such as lymphocyte and platelets need to be further analysed. Also for better generalization, the model needs to be further validated on other larger data sets and also on samples which include children, as the tested sample only consisted of adult patients and had only 11 DF patients.

## Conclusions

This study is an attempt to build a mathematical model, to address the combined effect of cytokines and immune mediators S1P, IL-1β, TNF-α, PAF and IL-10, and determine the severity of dengue at an early stage. We developed a mathematical model using fuzzy logic operators, Hamacher and OWA operators. Our model is different from a majority of previous studies as, rather than considering the individual effect of cytokines, the combined effect from several cytokines is considered.

The model performs well in 96, 108 and 120 h from onset of fever and performs best with an accuracy of 85% at 108 h from onset of fever. With the high accuracy level of the model it could be used as a useful asset to determine patients proceeding to DHF level at an early stage, and thereby to reduce the mortality rate and make optimal use of available resources. However, the model’s tendency to overestimate the risk of DF patients is a concern. Sensitivity analysis indicates that the model is robust.

## Additional files

Additional file 1: Theoretical Framework. (DOCX 20 kb)
Additional file 2: MATLAB code for model. (DOCX 34 kb)
Additional file 3: Validation Data. (XLSX 20 kb)

## Abbreviations

DF: Dengue fever
DHF: Dengue haemorrhagic fever
IL-10: Interleukin -10
IL-1β: Interleukin- 1β
PAF: Platelet activating factor
S1P: Sphingosine 1-phosphate
TNF-α: Tumor necrosis factor
