# Supplementary material for: Mathematical modelling and a systems science approach to describe the role of cytokines in the evolution of severe dengue
Source: BMC Syst Biol. 2017 Mar 11;11:34. doi: 10.1186/s12918-017-0415-3 (PMC5346240; doi:10.1186/s12918-017-0415-3)
Supplement: Additional file 1: — Theoretical Framework. (DOCX 20 kb) [file 12918_2017_415_MOESM1_ESM.docx]

**Additional File 1 (A1)**

**Theoretical Framework**

*Fuzzy Set*

Elements of a fuzzy set are mapped to a universe of membership values by a membership function, which maps elements of a fuzzy set *A* to a real number value in the interval [0, 1]. This mapping is given by $\mu_{A}\left( x \right)\in[0,1]$ and $\mu_{A}\left( x \right)$ refers to the degree of membership of element *x* in fuzzy set *A*. Elements with $\mu_{A}\left( x \right)=1$ and $\mu_{A}\left( x \right)=0$ represents, respectively, fully belongingness and not-belongingness of *x* in *A* while elements with $\mu_{A}\left( x \right)\in(0,1)$ represents partial membership of *x* in *A* [1].

*Trapezoidal Membership function*

The trapezoidal membership function of a vector *x* depends on four scalar parameters a, b, c, and d, which is given by

$$f\left( x;a,b,c,d \right)=\left\{ \begin{aligned} 0 ; x\leq a \\ \frac{x-a}{b-a} ; a\leq x\leq b \\ 1 ; b\leq x\leq c \\ \frac{d-x}{d-c}; c\leq x\leq d \\ 0 ; d\leq x \end{aligned} \right.$$

The parameters a and d locate the "feet" of the trapezoid and the parameters b and c locate the "shoulders" [2].

*Hamacher Operator*

$\mu_{H}\left( x \right)=\left\{ \begin{aligned} 0 ;if \mu_{A}\left( x \right)= \mu_{B}\left( x \right)=0 \\ \frac{\mu_{A}\left( x \right)\mu_{B}\left( x \right)}{\mu_{A}\left( x \right)+\mu_{B}\left( x \right)-\mu_{A}\left( x \right)\mu_{B}\left( x \right)} ;otherwise \end{aligned} \right.$ (1)

where $\mu_{A}\left( x \right)$*,* $\mu_{B}\left( x \right)$ are the membership function values of the fuzzy sets A and B respectively.

*Ordered Weighted Aggregation (OWA) Operator*

OWA operator is defined as, where $b_{j}$ is the j^th^ largest element of the collection of aggregated objects $a_{1}{,a}_{2}{,\ldots,a}_{n}$ and $\sum_{j=1}^{n} w_{\begin{aligned} j \\ \end{aligned}}=1$ and $w_{j}\in[0,1]$ [3].

$$OWA\left( a_{1},a_{2},\ldots.,a_{n} \right)=\sum_{j=1}^{n} w_{\begin{aligned} j \\ \end{aligned}}b_{j} (2)$$

Yager [4, 5] suggests a way to determine the weights of OWA operator by,

$w_{i}=Q\left( \frac{i}{n} \right)-Q\left( \frac{i-1}{n} \right) ;i=1,2,,\ldots,n$ (3)

and the non-decreasing quantifier Q is defined by Zadeh [6] as, given below, where$l,m,r\in[0,1]$.

$Q=\left\{ \begin{aligned} 0 ;if r<l \\ \frac{r-l}{m-l} ;if l\leq r\leq m \\ 1 ; if r>m \end{aligned} \right.$ (4)

‘Orness measure’ which measures the degree to which the aggregation operation is like an ‘OR’ operation is defined by Yager [4] as given below, which lies in the interval [0, 1].

$$orness\left( w \right)=\frac{1}{(n-1)} \sum_{i=1}^{n} \left( n-i \right)w_{i} (5)$$

*Concentration*

Concentration is a fuzzy logic concept that allows to further reduce the original membership values of the elements that are partly in the set [1]. This is defined as

$U_{CON\left( A \right)}\left( x \right)= {U^{\alpha}}_{A}\left( x \right) ; \alpha>1$ (6)

This allows us to allocate higher importance to the parameters that are vital to the model in determining disease severity.

**References**

| 1. Ross TJ. Fuzzy Logic with Engineering Applications, 3 rd ed. John Wiley & Sons Ltd; 2010. |
| --- |
| 1. Matlab. Fuzzy Logic Toolbox: Trapezoidal-shaped membership function (R2016a). http://in.mathworks.com/help/fuzzy/trapmf.html. Accessed 05 Jan 2016. 2. Xu Z. An overview of methods for determining OWA weights. Int J Intell Syst. 2005; 20:843-65. |
| 1. Yager RR. Families of OWA operators. Fuzzy Set Syst. 1993;59:125-48. |
| 1. Yager RR. On ordered weigted averaging aggregation operators in multicriteria decision making. IEEE Trans Man Cybern*.*1988:18:183-90. |
| 1. Zadeh L. A computational approach to fuzzy quantifiers in natural languages. Comput Math Appl. 1983;9:149-84. |
