# Supplementary material for: Mathematical modelling and a systems science approach to describe the role of cytokines in the evolution of severe dengue
Source: BMC Syst Biol. 2017 Mar 11;11:34. doi: 10.1186/s12918-017-0415-3 (PMC5346240; doi:10.1186/s12918-017-0415-3)
Supplement: Additional file 2: — MATLAB code for model. (DOCX 34 kb) [file 12918_2017_415_MOESM2_ESM.docx]

**Additional File 2 (MATLAB codes)**

**1. Model Development**

function f=modelValidate(PAF,IL10,IL1b,S1P,TNF)

%This function has the basic mathematical model and can be used for %validation and to determine patient severity level. The input %parameters are the row cytokine and inflammatory mediator values of %PAF,IL10,IL1b,S1P,TNF

%Fuzzifying the row values through each membership function.

%membershipPAF(),membershipIL110(),membershipIL1b(),membershipS1P,

%memmbershipIL1b are the membership functions of each parameter

%which takes as input, the row cytokine values of the patients.

%concentrate() function handles fuzzy concentration. PAF, IL10 and

%and TNF are concentrated by 1.2,1.1,1.1 respectively. It takes as

%input the fuzzified membership values and the amount in which it

% needs to be concentrated.

a=concentrate((membershipPAF(length(PAF),PAF)),1.2);

b=concentrate((membershipIL10(length(IL10),IL10)),1.1);

c=membershipIL1b(length(IL1b),IL1b);

d=membershipS1P(length(S1P),S1P);

e=concentrate((membershipTNF(length(TNF),TNF)),1.1);

%This function creates the ambiguous region

ambiguousRegionCreate()

%H1 takes the hamacher product between IL1b and S1P.hamacher() is a

% function which computes the Hamacher product defined in

%Additional File 1, equation (1)

H1=hamacher(c,d);

%H2 takes the hamacher product between IL10 and TNF

H2=hamacher(b,e);

%H3 takes the hamacher product between H2 and PAF

H3=hamacher(H2,a);

%the Hamacher products H1 and H3 are combined through the OWA

%operator.

owavalidate=0.4.*max(H1,H3)+0.6.*min(H1,H3);

%This is to plot the patients’ model value on the model regions to

%see which region the patient has fallen into. As in Fig.4, 5

%and Fig.6.

co=[0 0 0];

scatter3(H1,H3,owavalidate,[],co,'filled');

set(gcf,'renderer','painters')

set(gca,'drawmode','fast')

%outputs the final model result which measures the unfavourability

%to attain severe dengue.

f=owavalidate;

end

%concentrate() handles fuzzy concentration. It takes as

%input the fuzzified membership values and the amount in which it

% needs to be concentrated.

function f=concentrate(x,alpha)

f=[];

f=[f;x.^alpha];

end

%membershipPAF fuzzifies the row PAF value into respective membership %value. Takes as input the length of the vector of patient PAF values %and the vector which includes row PAF values of patients.

function f=membershipPAF(x,para)

yPAF=[];%used to hold each patient’s respective membership PAF

%value.

%The for loop runs through the length of the input vector. At each

%time taking a single patient’s row PAF value.

for n=1:x

%Calls for the PAFfunc which has the membership function

%defined in equation (5). It takes each patients row PAF value,

%and calculates the corresponding membership value. This

% membership value is then appended into yPAF.

yPAF=[yPAF;PAFfunc(para(n))];

end

%outputs the membership values of PAF.

f=yPAF;

end

%This function calculates the membership function values as defined in

%equation (5).

function f=PAFfunc(x)

if x<=10

f=1; %If the row PAF value is <=10, then the membership value=1

elseif x<=100

f=(100-x)/90; %If the row PAF value is <=100, membership PAF

% value is given by (100-x)/90

elseif x>100 %If the row PAF value is >100, membership PAF value is

% =0

f=0;

else

f=NaN; %This is to handle missing patient data values

end

end

%membershipIL10 fuzzifies the row IL10 value into respective membership %value. Same as the explanation in membershipPAF().

function f=membershipIL10(x,para)

yIL10=[];

for n=1:x

yIL10=[yIL10;IL10func(para(n))];

end

f=yIL10;

end

function f=IL10func(x)

if x<=20

f=1;

elseif x<=110

f=(110-x)/90;

elseif x>110

f=0;

else

f=NaN;

end

end

%membershipIL1b fuzzifies the row IL1b value into respective membership %value. Same as the explanation in membershipPAF().

function f=membershipIL1b(x,para)

yIL1b=[];

for n=1:x

yIL1b=[yIL1b;IL1bfunc(para(n))];

end

f=yIL1b;

end

function f=IL1bfunc(x)

if x<=30.5

f=1;

elseif x<=33.5

f=(33.5-x)/3;

elseif x>33.5

f=0;

else

f=NaN;

end

end

%membershipS1P fuzzifies the row S1P value into respective membership %value. Same as the explanation in membershipPAF().

function f=membershipS1P(x,para)

yS1P=[];

for n=1:x

yS1P=[yS1P;S1Pfunc(para(n))];

end

f=yS1P

end

function f=S1Pfunc(x)

if x<0.5

f=0;

elseif x<=1.5

f=x-0.5;

elseif x>1.5

f=1;

else

f=NaN;

end

end

%membershipTNF fuzzifies the row TNF value into respective membership %value. Same as the explanation in membershipPAF().

function f=membershipTNF(x,para)

yTNF=[];

for n=1:x

yTNF=[yTNF;TNFfunc(para(n))];

end

f=yTNF

end

function f=TNFfunc(x)

if x<=15

f=1;

elseif x<=30

f=(30-x)/15;

elseif x>30

f=0;

else

f=NaN;

end

end

%hamacher() is a function which computes the Hamacher product defined %in Additional File 1, equation (1). It takes two membership values as %input.x and y can be a vector of membership values of two cytokines.

function f=hamacher(x,y)

f = zeros(numel(x),1);

for j=1:numel(x)

if x(j)==0 && y(j)==0

f(j,1)=0;

else

f(j,1)=(x(j)*y(j))/(x(j)+y(j)-(x(j)*y(j)));

end

end

end

function ambiguousRegionCreate()

%In the ambiguous region it cannot be determined specifically whether

%the patient is DHF or DF.

H1values=[];

H2values=[];

H3values=[];

%S1Pval,IL1bval represents ambiguous levels of individual cytokines.

%These are determined using the cytokine values which resulted in

%around 0.5 degrees of membership values.

S1Pval=0.9:0.01:1.2;

IL1bval=30.7:0.05:31;

%These S1P and IL1b individual ambiguous levels are converted into

% membership values.

x=membershipS1P(length(S1Pval),S1Pval);

y=membershipIL1b(length(IL1bval),IL1bval);

x=x';

y=y';

for i=1:length(x)%for each value in S1Pval

for j=1:length(y) %for each value in IL1bval

z=hamacher(x(i),y(j));%For each S1P and IL1b value that lie in

%individual ambiguity level, the hamacher product is considered

%to calculate the ambiguous level resulting from combined S1P

%and IL1b.

H1values=[H1values z]; %The hamacher product results are stored

% in H1values vector.

end

end

%Once the hamacher product of all possible combinations of combined

%ambiguity resulting from both S1P and IL1b is evaluated, the minimum

%and maximum of these values are considered to build the lower and

%upper limit of the ambiguous region.

H1min= min(H1values);

H1max= max(H1values);

%TNFval,IL10val represents ambiguous levels of individual cytokines.

%These are determined using the cytokine values which resulted in

%around 0.5 degrees of membership values.

TNFval=17.5:0.5:19;

IL10val=38:0.5:40.5;

%These TNF and IL10 individual ambiguous levels are converted into

%membership values and are then concentrated by an amount of 1.1

p1=membershipTNF(length(TNFval),TNval);

q1=membershipIL10(length(IL10val),IL10val);

p=concentrate(p1,1.1);

q=concentrate(q1,1.1);

for i=1:length(p)

for j=1:length(q)

%For each TNF and IL10 value that lie in

%individual ambiguity level, the hamacher product is considered

%to calculate the ambiguous level resulting from combined TNF

%and IL10.

z2=hamacher(p(i),q(j));

%The hamacher product results are stored in H2values vector.

H2values=[H2values z2];

end

end

%PAFval represents ambiguous level of individual cytokine PAF.

%These are determined using the cytokine values which resulted in

%around 0.5 degrees of membership values.

PAFval=48:0.5:50;

%This PAF individual ambiguous level is converted into

%membership values and then concentrated by an amount of 1.2

r1=membershipPAF(length(PAFval),PAFval);

r=concentrate(r1,1.2);

for i=1:length(H2values)

for j=1:length(r)

%For each H2values and PAF value that lie in

%individual ambiguity level, the hamacher product is considered

%to calculate the ambiguous level resulting from combined TNF

%IL10 and PAF.

z3=hamacher(H2values(i),r(j));

%The hamacher product results are stored in H3values vector.

H3values=[H3values z3];

end

end

%Once the hamacher product of all possible combinations of combined

%ambiguity resulting from TNF,IL10 and PAF is evaluated, the minimum

%and maximum of these values are considered to build the lower and

%upper limit of the ambiguous region.

H3min=min(H3values);

H3max=max(H3values);

ambiguousLower=0.4*max(H1min,H3min)+0.6*min(H1min,H3min);

ambiguousUpper=0.4*max(H1max,H3max)+0.6*min(H1max,H3max);

%To create a plot which shows the DHF,DF and ambiguous region. This is

% drawn with Hamacher product resulting from S1P and IL1b (H1) being

%in the x axis and Hamacher product resulting from TNF,IL10 and PAF

%(H2)being in the y axis and the resulting OWA value which measures the

%final model result for dengue severity being in the z axis.

%As theoretically H1 and H2 takes values in [0,1], x1 and y1 represent

%these possible values.

x1=0:0.01:1;

y1=0:0.01:1;

[X,Y]=meshgrid(x1,y1);

%calculates the OWA value for all the theoretical possibilities of H1

%and H2.

Z=0.4.*max(X,Y)+0.6.*min(X,Y);

surf(X,Y,Z);

xlabel('H1')

ylabel('H2')

zlabel('OWA operator value')

hold on;

%Plotting the theoretically created ambiguous region against H1 and H2.

%Any OWA value lies in between [0,1]. So all combinations in [0,1] are

%considered.

for i=0:0.01:1

for j=0:0.01:1

zval=0.4.*max(i,j)+0.6.*min(i,j);

%Any OWA value that lies in between the theoretically

%constructed lower and upper limits of the ambiguous region

%creates the overall ambiguous region of the model

if(zval>=ambiguousLower && zval<=ambiguousUpper)

plot3(i,j,zval,'.','color','w'); %ambiguous region is

%plotted in white

end

end

end

hold on;

end

**2. Accuracy**

%calculates the model accuracy

%Takes as input the final model result which measures the %unfavourability to attain severe dengue. Model results for both DHF %and DF patients are considered.

function f = accuracy(DHFmodelValues,DFmodelValues)

%DHFcount has the correctly classified DHF patients. It is

%calculated by calling the DHFaccuracy() function.

DHFcount=DHFaccuracy(DHFmodelValues);

%DFcount has the correctly classified DF patients. It is

%calculated by calling the DFaccuracy() function.

DFcount=DFaccuracy(DFmodelValues);

%final accuracy of the model f=(DHFcount+DFcount)/(length(DHFmodelValues)+length(DFmodelValues))*100;

end

%DHFaccuracy() takes as input the final model values of the patients %who are determined as DHF through medical diagnosis.

function f= DHFaccuracy(DHFvalues)

count=0; %counts the correctly classifications of DHF patients

for i=1:length(DHFvalues)%loops through each of the final model

%results of DHF patients

if DHFvalues(i)<=0.51

%As the unfavourability to attain severe dengue is measured and the %upper ambiguous level that was calculated theoretically was found to %be 0.51, those that fall below this value are DHF patients. So, if it %is true that the model value is <=0.51 then it is a correct %classification of the DHF patient as the model decision agrees with %the medical decision.

count=count+1;

end

end

f=count;

end

%DFaccuracy() takes as input the final model values of the patients %who are determined as DF through medical diagnosis.

function f= DFaccuracy(DFvalues)

count=0; %counts the correctly classifications of DF patients

for i=1:length(DFvalues)

%As the unfavourability to attain severe dengue is measured and the %lower ambiguous level that was calculated theoretically was found to %be 0.36, those that fall above this value are DF patients. So, if it %is true that the model value is >=0.36 then it is a correct %classification of the DF patient as the model decision agrees with %the medical decision.

if DFvalues(i)>=0.36

count=count+1;

end

end

f=count;

end

**3. Sensitivity**

%Sensitivity analysis when the boundary values of each of the %membership functions are changed by a small amount. The change in the %ambiguous region is analysed. The existing ambiguous region has a %lower limit of 0.36 and upper limit of 0.51. Since, the sensitivity %looks into the change in the ambiguous region, these functions are %similar to that of ambiguousRegionCreate() and only the parameter in %which the sensitivity analysed is varied from an amount ‘delta’

%sensitivity at lower cut off value of PAF

function f=ambiguousRegionSensitivityPAFlower()

H1values=[];

H2values=[];

H3values=[];

S1Pval=0.9:0.01:1.2;

IL1bval=30.7:0.05:31;

y=membershipIL1b(length(IL1bval),IL1bval);

y=y';

x=membershipS1P(length(S1Pval),S1Pval);

x=x';

for i=1:length(x)

for j=1:length(y)

z=hamacher(x(i),y(j));

H1values=[H1values z];

end

end

H1min=min(H1values);

H1max=max(H1values);

TNFval=17.5:0.5:19;

IL10val=38:0.5:40.5;

q1=membershipIL10(length(IL10val),IL10val);

q=concentrate(q1,1.1);

p1=membershipTNF(length(TNFval),TNFval);

p=concentrate(p1,1.1);

for i=1:length(p)

for j=1:length(q)

z2=hamacher(p(i),q(j));

H2values=[H2values z2];

end

end

H3min=[];

H3max=[];

PAFval=48:0.5:50;

%The lower PAF cut off value used in the model is 10. Sensitivity

%is checked when this value is changed between 7-13.

PAFdelta=[-3 -2 -1 0 1 2 3];

PAFlower=[7 8 9 10 11 12 13];

for m=1:length(PAFdelta)

r1=membershipPAFlower(length(PAFval),PAFval,PAFdelta(m));

r=concentrate(r1,1.2);

for s=1:length(H2values)

for t=1:length(r)

z3=hamacher(H2values(s),r(t));

H3values=[H3values z3];

end

end

H3valmax=max(H3values);

H3max=[H3max H3valmax];

H3valmin=min(H3values);

H3min=[H3min H3valmin];

H3values=[];

end

ambiguousLower=[];

ambiguousUpper=[];

for n=1:length(PAFdelta) %for each change in the PAF lower level

%The lower level of the ambiguous region is calculated and is

% appended to ambiguousLower

Lower=0.4*max(H1min,H3min(n))+0.6*min(H1min,H3min(n));

ambiguousLower=[ambiguousLower Lower];

%The upper level of the ambiguous region is calculated and is

% appended to ambiguousUpper

Upper=0.4*max(H1max,H3max(n))+0.6*min(H1max,H3max(n));

ambiguousUpper=[ambiguousUpper Upper];

end

display(ambiguousLower) ;

display(ambiguousUpper);

%The change of the lower level of the ambiguous region when the

% lower cut off value of the membership function of PAF is changed

%is plotted in blue

plot(PAFlower,ambiguousLower,'b--o','MarkerSize',5,'MarkerFaceColor','b','LineWidth',2);

hold on;

%The change of the upper level of the ambiguous region when the

%lower cut off value of the membership function of PAF is changed

%is plotted in red

plot(PAFlower,ambiguousUpper,'r--o','MarkerSize',5,'MarkerFaceColor','r','LineWidth',2);

f=ambiguousUpper;

end

%membershipPAFlower() calculates the membership values when the lower %cut of point of PAF is changed by an amount of delta. X refers to the %length of the row PAF levels that are to be converted to membership %values. Para refers to the row PAF levels and delta refers to the %amount by which we change the lower cut of value of the PAF membership %function

function f=membershipPAFlower(x,para,delta)

yPAF=[];

for n=1:x

yPAF=[yPAF;PAFfuncLower(para(n),delta)];

end

f=yPAF;

end

%PAFfunclower() is the changed membership function due to the change in %the lower cut off value by an amount of delta. This is derived by %changing the lower cut off value of 10 by an amount delta in the %equation (5)

function f=PAFfuncLower(x,delta)

if x<=10+delta

f=1;

elseif x<=100

f=(x-100)/((10+delta)-100);

elseif x>100

f=0;

else

f=NaN;

end

end

%sensitivity at upper cut off value of PAF

function f=ambiguousRegionSensitivityPAFupper()

H1values=[];

H2values=[];

H3values=[];

S1Pval=0.9:0.01:1.2;

IL1bval=30.7:0.05:31;

y=membershipIL1b(length(IL1bval),IL1bval);

y=y';

x=membershipS1P(length(S1Pval),S1Pval);

x=x';

for i=1:length(x)

for j=1:length(y)

z=hamacher(x(i),y(j));

H1values=[H1values z];

end

end

H1min=min(H1values);

H1max=max(H1values);

TNFval=17.5:0.5:19;

IL10val=38:0.5:40.5;

q1=membershipIL10(length(IL10val),IL10val);

q=concentrate(q1,1.1);

p1=membershipTNF(length(TNFval),TNFval);

p=concentrate(p1,1.1);

for i=1:length(p)

for j=1:length(q)

z2=hamacher(p(i),q(j));

H2values=[H2values z2];

end

end

H3min=[];

H3max=[];

PAFval=48:0.5:50;

%The upper PAF cut off value used in the model is 100. Sensitivity

%is checked when this value is changed between 97-103.

PAFdelta=[-3 -2 -1 0 1 2 3];

PAFupper=[97 98 99 100 101 102 103];

for m=1:length(PAFdelta)

r1=membershipPAFupper(length(PAFval),PAFval,PAFdelta(m));

r=concentrate(r1,1.2);

for s=1:length(H2values)

for t=1:length(r)

z3=hamacher(H2values(s),r(t));

H3values=[H3values z3];

end

end

H3valmax=max(H3values);

H3max=[H3max H3valmax];

H3valmin=min(H3values);

H3min=[H3min H3valmin];

H3values=[];

end

ambiguousLower=[];

ambiguousUpper=[];

for n=1:length(PAFdelta)

Lower=0.4*max(H1min,H3min(n))+0.6*min(H1min,H3min(n));

ambiguousLower=[ambiguousLower Lower];

Upper=0.4*max(H1max,H3max(n))+0.6*min(H1max,H3max(n));

ambiguousUpper=[ambiguousUpper Upper];

end

display(ambiguousLower) ;

display(ambiguousUpper);

plot(PAFupper,ambiguousLower,'b--o','MarkerSize',5,'MarkerFaceColor','b','LineWidth',2);

hold on;

plot(PAFupper,ambiguousUpper,'r--o','MarkerSize',5,'MarkerFaceColor','r','LineWidth',2);

f=ambiguousUpper;

end

function f=membershipPAFupper(x,para,delta)

yPAF=[];

for n=1:x

yPAF=[yPAF;PAFfuncUpper(para(n),delta)];

end

f=yPAF;

end

%PAFfuncupper() is the changed membership function due to the change in %the upper cut off value by an amount of delta. This is derived by %changing the upper cut off value of 100 by an amount delta in the %equation (5)

function f=PAFfuncUpper(x,delta)

if x<=10

f=1;

elseif x<=100+delta

f=(x-(100+delta))/(10-(100+delta));

elseif x>100+delta

f=0;

else

f=NaN;

end

end

% sensitivity at IL1b lower cut off value

function f=ambiguousRegionSensitivityIL1blower()

H1values=[];

H2values=[];

H3values=[];

S1Pval=0.9:0.01:1.2;

IL1bval=30.7:0.05:31;

x=membershipS1P(length(S1Pval),S1Pval);

x=x';

IL1bdelta=[-0.03 -0.02 -0.01 0 0.01 0.02 0.03];

IL1blower=[30.47 30.48 30.49 30.5 30.51 30.52 30.53];

H1min=[];

H1max=[];

for m=1:length(IL1bdelta)

y=membershipIL1blower(length(IL1bval),IL1bval,IL1bdelta(m));

y=y';

for i=1:length(x)

for j=1:length(y)

z=hamacher(x(i),y(j));

H1values=[H1values z];

end

end

H1valmax=max(H1values);

H1max=[H1max H1valmax];

H1valmin=min(H1values);

H1min=[H1min H1valmin];

H1values=[];

end

TNFval=17.5:0.5:19;

IL10val=38:0.5:40.5;

p1=membershipTNF(length(TNFval),TNFval);

q1=membershipIL10(length(IL10val),IL10val);

p=concentrate(p1,1.1);

q=concentrate(q1,1.1);

for i=1:length(p)

for j=1:length(q)

z2=hamacher(p(i),q(j));

H2values=[H2values z2];

end

end

PAFval=48:0.5:50;

r1=membershipPAF(length(PAFval),PAFval);

r=concentrate(r1,1.2);

for i=1:length(H2values)

for j=1:length(r)

z3=hamacher(H2values(i),r(j));

H3values=[H3values z3];

end

end

ambiguousLower=[];

ambiguousUpper=[];

H3min=min(H3values);

H3max=max(H3values);

for i=1:length(IL1bdelta)

Lower=0.4.*max(H1min(i),H3min)+0.6.*min(H1min(i),H3min);

ambiguousLower=[ambiguousLower Lower];

Upper=0.4.*max(H1max(i),H3max)+0.6.*min(H1max(i),H3max);

ambiguousUpper=[ambiguousUpper Upper];

end

display(ambiguousLower) ;

display(ambiguousUpper);

plot(IL1blower,ambiguousLower,'b--o','MarkerSize',5,'MarkerFaceColor','b','LineWidth',2);

hold on;

plot(IL1blower,ambiguousUpper,'r--o','MarkerSize',5,'MarkerFaceColor','r','LineWidth',2);

f=ambiguousUpper;

end

function f=membershipIL1blower(x,para,delta)

yIL1b=[];

for n=1:x

yIL1b=[yIL1b;IL1bfuncLower(para(n),delta)];

end

f=yIL1b;

end

%IL1bfuncLower() is the changed membership function due to the change %in the lower cut off value by an amount of delta. This is derived by %changing the lower cut off value of 30.5 by an amount delta in the %equation (2)

function f=IL1bfuncLower(x,delta)

if x<=30.5+delta

f=1;

elseif x<=33.5

f=(x-33.5)/((30.5+delta)-33.5);

elseif x>33.5

f=0;

else

f=NaN;

end

end

%Sensitivity at IL1b upper cut off

function f=ambiguousRegionSensitivityIL1bupper()

H1values=[];

H2values=[];

H3values=[];

S1Pval=0.9:0.01:1.2;

IL1bval=30.7:0.05:31;

x=membershipS1P(length(S1Pval),S1Pval);

x=x';

IL1bdelta=[-0.03 -0.02 -0.01 0 0.01 0.02 0.03];

IL1bupper=[33.47 33.48 33.49 33.5 33.51 33.52 33.53];

H1min=[];

H1max=[];

for m=1:length(IL1bdelta)

y=membershipIL1bupper(length(IL1bval),IL1bval,IL1bdelta(m));

y=y';

for i=1:length(x)

for j=1:length(y)

z=hamacher(x(i),y(j));

H1values=[H1values z];

end

end

H1valmax=max(H1values);

H1max=[H1max H1valmax];

H1valmin=min(H1values);

H1min=[H1min H1valmin];

H1values=[];

end

TNFval=17.5:0.5:19;

IL10val=38:0.5:40.5;

p1=membershipTNF(length(TNFval),TNFval);

q1=membershipIL10(length(IL10val),IL10val);

p=concentrate(p1,1.1);

q=concentrate(q1,1.1);

for i=1:length(p)

for j=1:length(q)

z2=hamacher(p(i),q(j));

H2values=[H2values z2];

end

end

PAFval=48:0.5:50;

r1=membershipPAF(length(PAFval),PAFval);

r=concentrate(r1,1.2);

for i=1:length(H2values)

for j=1:length(r)

z3=hamacher(H2values(i),r(j));

H3values=[H3values z3];

end

end

ambiguousLower=[];

ambiguousUpper=[];

H3min=min(H3values);

H3max=max(H3values);

for i=1:length(IL1bdelta)

Lower=0.4.*max(H1min(i),H3min)+0.6.*min(H1min(i),H3min);

ambiguousLower=[ambiguousLower Lower];

Upper=0.4.*max(H1max(i),H3max)+0.6.*min(H1max(i),H3max);

ambiguousUpper=[ambiguousUpper Upper];

end

display(ambiguousLower) ;

display(ambiguousUpper);

plot(IL1bupper,ambiguousLower,'b--o','MarkerSize',5,'MarkerFaceColor','b','LineWidth',2);

hold on;

plot(IL1bupper,ambiguousUpper,'r--o','MarkerSize',5,'MarkerFaceColor','r','LineWidth',2);

f=ambiguousUpper;

end

function f=membershipIL1bupper(x,para,delta)

yIL1b=[];

for n=1:x

yIL1b=[yIL1b;IL1bfuncUpper(para(n),delta)];

end

f=yIL1b;

end

%IL1bfuncUpper() is the changed membership function due to the change %in the upper cut off value by an amount of delta. This is derived by %changing the upper cut off value of 33.5 by an amount delta in the %equation (8)

function f=IL1bfuncUpper(x,delta)

if x<=30.5

f=1;

elseif x<=33.5+delta

f=(x-(33.5+delta))/(30.5-(33.5+delta));

elseif x>33.5+delta

f=0;

else

f=NaN;

end

end

%IL-10 lower cut off value

function f=ambiguousRegionSensitivityIL10lower()

H1values=[];

H2values=[];

H3values=[];

S1Pval=0.9:0.01:1.2;

IL1bval=30.7:0.05:31;

y=membershipIL1b(length(IL1bval),IL1bval);

y=y';

x=membershipS1P(length(S1Pval),S1Pval);

x=x';

for i=1:length(x)

for j=1:length(y)

z=hamacher(x(i),y(j));

H1values=[H1values z];

end

end

H1min=min(H1values);

H1max=max(H1values);

TNFval=17.5:0.5:19;

IL10val=38:0.5:40.5;

p1=membershipTNF(length(TNFval),TNFval);

p=concentrate(p1,1.1);

IL10delta=[-3 -2 -1 0 1 2 3];

IL10lower=[17 18 19 20 21 22 23];

H3min=[];

H3max=[];

PAFval=48:0.5:50;

r1=membershipPAF(length(PAFval),PAFval);

r=concentrate(r1,1.2);

for m=1:length(IL10delta)

q1=membershipIL10lower(length(IL10val),IL10val,IL10delta(m));

q=concentrate(q1,1.1);

for i=1:length(p)

for j=1:length(q)

z2=hamacher(p(i),q(j));

H2values=[H2values z2];

end

end

for s=1:length(H2values)

for t=1:length(r)

z3=hamacher(H2values(s),r(t));

H3values=[H3values z3];

end

end

H3valmax=max(H3values);

H3max=[H3max H3valmax];

H3valmin=min(H3values);

H3min=[H3min H3valmin];

H2values=[];

H3values=[];

end

ambiguousLower=[];

ambiguousUpper=[];

for n=1:length(IL10delta)

Lower=0.4*max(H1min,H3min(n))+0.6*min(H1min,H3min(n));

ambiguousLower=[ambiguousLower Lower];

Upper=0.4*max(H1max,H3max(n))+0.6*min(H1max,H3max(n));

ambiguousUpper=[ambiguousUpper Upper];

end

display(ambiguousLower) ;

display(ambiguousUpper);

plot(IL10lower,ambiguousLower,'b--o','MarkerSize',5,'MarkerFaceColor','b','LineWidth',2);

hold on;

plot(IL10lower,ambiguousUpper,'r--o','MarkerSize',5,'MarkerFaceColor','r','LineWidth',2);

f=ambiguousUpper;

end

function f=membershipIL10lower(x,para,delta)

yIL10=[];

for n=1:x

yIL10=[yIL10;IL10funcLower(para(n),delta)];

end

f=yIL10;

end

%IL10funcLower() is the changed membership function due to the change %in the lower cut off value by an amount of delta. This is derived by %changing the lower cut off value of 20 by an amount delta in the %equation (3)

function f=IL10funcLower(x,delta)

if x<=20+delta

f=1;

elseif x<=110

f=(x-110)/((20+delta)-110);

elseif x>110

f=0;

else

f=NaN;

end

end

%Sensitivity at IL10 upper cut off value

function f=ambiguousRegionSensitivityIL10upper()

H1values=[];

H2values=[];

H3values=[];

S1Pval=0.9:0.01:1.2;

IL1bval=30.7:0.05:31;

y=membershipIL1b(length(IL1bval),IL1bval);

y=y';

x=membershipS1P(length(S1Pval),S1Pval);

x=x';

for i=1:length(x)

for j=1:length(y)

z=hamacher(x(i),y(j));

H1values=[H1values z];

end

end

H1min=min(H1values);

H1max=max(H1values);

TNFval=17.5:0.5:19;

IL10val=38:0.5:40.5;

p1=membershipTNF(length(TNFval),TNFval);

p=concentrate(p1,1.1);

IL10delta=[-7.5 -5 -2.5 0 2.5 5 7.5 ];

IL10upper=[102.5 105 107.5 110 112.5 115 117.5];

H3min=[];

H3max=[];

PAFval=48:0.5:50;

r1=membershipPAF(length(PAFval),PAFval);

r=concentrate(r1,1.2);

for m=1:length(IL10delta)

q1=membershipIL10upper(length(IL10val),IL10val,IL10delta(m));

q=concentrate(q1,1.1);

for i=1:length(p)

for j=1:length(q)

%z2=(p(i)*q(j))/(p(i)+q(j)-(p(i)*q(j)));

z2=hamacher(p(i),q(j));

H2values=[H2values z2];

end

end

for s=1:length(H2values)

for t=1:length(r)

z3=hamacher(H2values(s),r(t));

H3values=[H3values z3];

end

end

H3valmax=max(H3values);

H3max=[H3max H3valmax];

H3valmin=min(H3values);

H3min=[H3min H3valmin];

H2values=[];

H3values=[];

end

ambiguousLower=[];

ambiguousUpper=[];

for n=1:length(IL10delta)

Lower=0.4*max(H1min,H3min(n))+0.6*min(H1min,H3min(n));

ambiguousLower=[ambiguousLower Lower];

Upper=0.4*max(H1max,H3max(n))+0.6*min(H1max,H3max(n));

ambiguousUpper=[ambiguousUpper Upper];

end

display(ambiguousLower) ;

display(ambiguousUpper);

plot(IL10upper,ambiguousLower,'b--o','MarkerSize',5,'MarkerFaceColor','b','LineWidth',2);

hold on;

plot(IL10upper,ambiguousUpper,'r--o','MarkerSize',5,'MarkerFaceColor','r','LineWidth',2);

f=ambiguousUpper;

end

function f=membershipIL10upper(x,para,delta)

yIL10=[];

for n=1:x

yIL10=[yIL10;IL10funcUpper(para(n),delta)];

end

f=yIL10;

end

%IL10funcUpper() is the changed membership function due to the change %in the upper cut off value by an amount of delta. This is derived by %changing the upper cut off value of 110 by an amount delta in the %equation (3)

function f=IL10funcUpper(x,delta)

if x<=20

f=1;

elseif x<=110+delta

f=(x-(110+delta))/(20-(110+delta));

elseif x>110+delta

f=0;

else

f=NaN;

end

end

%TNF lower cut off value

function f=ambiguousRegionSensitivityTNFlower()

H1values=[];

H2values=[];

H3values=[];

S1Pval=0.9:0.01:1.2;

IL1bval=30.7:0.05:31;

y=membershipIL1b(length(IL1bval),IL1bval);

y=y';

x=membershipS1P(length(S1Pval),S1Pval);

x=x';

for i=1:length(x)

for j=1:length(y)

z=hamacher(x(i),y(j));

H1values=[H1values z];

end

end

H1min=min(H1values);

H1max=max(H1values);

TNFval=17.5:0.5:19;

IL10val=38:0.5:40.5;

q1=membershipIL10(length(IL10val),IL10val);

q=concentrate(q1,1.1);

TNFdelta=[-0.5 -0.25 0 0.25 0.5];

TNFlower=[14.5 14.75 15 15.25 15.5]

H3min=[];

H3max=[];

PAFval=48:0.5:50;

r1=membershipPAF(length(PAFval),PAFval);

r=concentrate(r1,1.2);

for m=1:5

p1=membershipTNFlower(length(TNFval),TNFval,TNFdelta(m));

p=concentrate(p1,1.1);

for i=1:length(p)

for j=1:length(q)

%z2=(p(i)*q(j))/(p(i)+q(j)-(p(i)*q(j)));

z2=hamacher(p(i),q(j));

H2values=[H2values z2];

end

end

for s=1:length(H2values)

for t=1:length(r)

z3=hamacher(H2values(s),r(t));

H3values=[H3values z3];

end

end

H3valmax=max(H3values);

H3max=[H3max H3valmax];

H3valmin=min(H3values);

H3min=[H3min H3valmin];

H2values=[];

H3values=[];

end

ambiguousLower=[];

ambiguousUpper=[];

for n=1:5

Lower=0.4*max(H1min,H3min(n))+0.6*min(H1min,H3min(n));

ambiguousLower=[ambiguousLower Lower];

Upper=0.4*max(H1max,H3max(n))+0.6*min(H1max,H3max(n));

ambiguousUpper=[ambiguousUpper Upper];

end

display(ambiguousLower) ;

display(ambiguousUpper);

plot(TNFlower,ambiguousLower,'b--o','MarkerSize',5,'MarkerFaceColor','b','LineWidth',2);

hold on;

plot(TNFlower,ambiguousUpper,'r--o','MarkerSize',5,'MarkerFaceColor','r','LineWidth',2);

f=ambiguousUpper;

end

function f=membershipTNFlower(x,para,delta)

yTNF=[];

for n=1:x

yTNF=[yTNF;TNFfuncLower(para(n),delta)];

end

f=yTNF

end

%TNFfuncLower() is the changed membership function due to the change %in the lower cut off value by an amount of delta. This is derived by %changing the lower cut off value of 15 by an amount delta in the %equation (4)

function f=TNFfuncLower(x,delta)

if x<=15+delta

f=1;

elseif x<=30

f=(x-30)/((15+delta)-30);

elseif x>30

f=0;

else

f=NaN;

end

end

%TNF upper cut off

function f=ambiguousRegionSensitivityTNFupper()

H1values=[];

H2values=[];

H3values=[];

S1Pval=0.9:0.01:1.2;

IL1bval=30.7:0.05:31;

y=membershipIL1b(length(IL1bval),IL1bval);

y=y';

x=membershipS1P(length(S1Pval),S1Pval);

x=x';

for i=1:length(x)

for j=1:length(y)

z=hamacher(x(i),y(j));

H1values=[H1values z];

end

end

H1min=min(H1values);

H1max=max(H1values);

TNFval=17.5:0.5:19;

IL10val=38:0.5:40.5;

q1=membershipIL10(length(IL10val),IL10val);

q=concentrate(q1,1.1);

TNFdelta=[-1 -0.75 -0.5 -0.25 0 0.25 0.5 0.75 1];

TNFupper=[29 29.25 29.5 29.75 30 30.25 30.5 30.75 31 ];

H3min=[];

H3max=[];

PAFval=48:0.5:50;

r1=membershipPAF(length(PAFval),PAFval);

r=concentrate(r1,1.2);

for m=1:9

p1=membershipTNFupper(length(TNFval),TNFval,TNFdelta(m));

p=concentrate(p1,1.1);

for i=1:length(p)

for j=1:length(q)

z2=hamacher(p(i),q(j));

H2values=[H2values z2];

end

end

for s=1:length(H2values)

for t=1:length(r)

z3=hamacher(H2values(s),r(t));

H3values=[H3values z3];

end

end

H3valmax=max(H3values);

H3max=[H3max H3valmax];

H3valmin=min(H3values);

H3min=[H3min H3valmin];

H2values=[];

H3values=[];

end

ambiguousLower=[];

ambiguousUpper=[];

for n=1:9

Lower=0.4*max(H1min,H3min(n))+0.6*min(H1min,H3min(n));

ambiguousLower=[ambiguousLower Lower];

Upper=0.4*max(H1max,H3max(n))+0.6*min(H1max,H3max(n));

ambiguousUpper=[ambiguousUpper Upper];

end

display(ambiguousLower) ;

display(ambiguousUpper);

plot(TNFupper,ambiguousLower,'b--o','MarkerSize',5,'MarkerFaceColor','b','LineWidth',2);

hold on;

plot(TNFupper,ambiguousUpper,'r--o','MarkerSize',5,'MarkerFaceColor','r','LineWidth',2);

f=ambiguousUpper;

end

function f=membershipTNFupper(x,para,delta)

yTNF=[];

for n=1:x

yTNF=[yTNF;TNFfuncUpper(para(n),delta)];

end

f=yTNF

end

%TNFfuncUpper() is the changed membership function due to the change %in the upper cut off value by an amount of delta. This is derived by %changing the upper cut off value of 30 by an amount delta in the %equation (4)

function f=TNFfuncUpper(x,delta)

if x<=15

f=1;

elseif x<=30+delta

f=(x-(30+delta))/(15-(30+delta));

elseif x>30+delta

f=0;

else

f=NaN;

end

end

%Sensitivity at S1P lower cut off value

function f=ambiguousRegionSensitivityS1Plower()

H1values=[];

H2values=[];

H3values=[];

S1Pval=0.9:0.01:1.2;

IL1bval=30.7:0.05:31;

y=membershipIL1b(length(IL1bval),IL1bval);

y=y';

S1Pdelta=[-0.03 -0.02 -0.01 0 0.01 0.02 0.03];

S1Plower=[0.47 0.48 0.49 0.5 0.51 0.52 0.53];

H1min=[];

H1max=[];

for m=1:length(S1Pdelta)

x=membershipS1Plower(length(S1Pval),S1Pval,S1Pdelta(m));

x=x';

for i=1:length(x)

for j=1:length(y)

z=hamacher(x(i),y(j));

H1values=[H1values z];

end

end

H1valmax=max(H1values);

H1max=[H1max H1valmax];

H1valmin=min(H1values);

H1min=[H1min H1valmin];

H1values=[];

end

TNFval=17.5:0.5:19;

IL10val=38:0.5:40.5;

p1=membershipTNF(length(TNFval),TNFval);

q1=membershipIL10(length(IL10val),IL10val);

p=concentrate(p1,1.1);

q=concentrate(q1,1.1);

for i=1:length(p)

for j=1:length(q)

z2=hamacher(p(i),q(j));

H2values=[H2values z2];

end

end

PAFval=48:0.5:50;

r1=membershipPAF(length(PAFval),PAFval);

r=concentrate(r1,1.2);

for i=1:length(H2values)

for j=1:length(r)

z3=hamacher(H2values(i),r(j));

H3values=[H3values z3];

end

end

ambiguousLower=[];

ambiguousUpper=[];

H3min=min(H3values);

H3max=max(H3values);

for i=1:length(S1Pdelta)

Lower=0.4.*max(H1min(i),H3min)+0.6.*min(H1min(i),H3min);

ambiguousLower=[ambiguousLower Lower];

Upper=0.4.*max(H1max(i),H3max)+0.6.*min(H1max(i),H3max);

ambiguousUpper=[ambiguousUpper Upper];

end

display(ambiguousLower) ;

display(ambiguousUpper);

plot(S1Plower,ambiguousLower,'b--o','MarkerSize',5,'MarkerFaceColor','b','LineWidth',2);

hold on;

plot(S1Plower,ambiguousUpper,'r--o','MarkerSize',5,'MarkerFaceColor','r','LineWidth',2);

f=ambiguousUpper;

end

function f=membershipS1Plower(x,para,delta)

yS1P=[];

for n=1:x

yS1P=[yS1P;S1PfuncLower(para(n),delta)];

end

f=yS1P

end

%S1PfuncLower() is the changed membership function due to the change %in the lower cut off value by an amount of delta. This is derived by %changing the lower cut off value of 0.5 by an amount delta in the %equation (1)

function f=S1PfuncLower(x,delta)

if x<0.5+delta

f=0;

elseif x<=1.5

f=(x-(0.5+delta))/(1.5-(0.5+delta));

elseif x>1.5

f=1;

else

f=NaN;

end

end

%S1P upper cut off value

function f=ambiguousRegionSensitivityS1Pupper()

H1values=[];

H2values=[];

H3values=[];

S1Pval=0.9:0.01:1.2;

IL1bval=30.7:0.05:31;

y=membershipIL1b(length(IL1bval),IL1bval);

y=y';

S1Pdelta=[-0.03 -0.02 -0.01 0 0.01 0.02 0.03];

S1Pupper=[1.47 1.48 1.49 1.5 1.51 1.52 1.53];

H1min=[];

H1max=[];

for m=1:length(S1Pdelta)

x=membershipS1Pupper(length(S1Pval),S1Pval,S1Pdelta(m));

x=x';

for i=1:length(x)

for j=1:length(y)

z=hamacher(x(i),y(j));

H1values=[H1values z];

end

end

H1valmax=max(H1values);

H1max=[H1max H1valmax];

H1valmin=min(H1values);

H1min=[H1min H1valmin];

H1values=[];

end

TNFval=17.5:0.5:19;

IL10val=38:0.5:40.5;

p1=membershipTNF(length(TNFval),TNFval);

q1=membershipIL10(length(IL10val),IL10val);

p=concentrate(p1,1.1);

q=concentrate(q1,1.1);

for i=1:length(p)

for j=1:length(q)

z2=hamacher(p(i),q(j));

H2values=[H2values z2];

end

end

PAFval=48:0.5:50;

r1=membershipPAF(length(PAFval),PAFval);

r=concentrate(r1,1.2);

for i=1:length(H2values)

for j=1:length(r)

z3=hamacher(H2values(i),r(j));

H3values=[H3values z3];

end

end

ambiguousLower=[];

ambiguousUpper=[];

H3min=min(H3values);

H3max=max(H3values);

for i=1:length(S1Pdelta)

Lower=0.4.*max(H1min(i),H3min)+0.6.*min(H1min(i),H3min);

ambiguousLower=[ambiguousLower Lower];

Upper=0.4.*max(H1max(i),H3max)+0.6.*min(H1max(i),H3max);

ambiguousUpper=[ambiguousUpper Upper];

end

display(ambiguousLower) ;

display(ambiguousUpper);

plot(S1Pupper,ambiguousLower,'b--o','MarkerSize',5,'MarkerFaceColor','b','LineWidth',2);

hold on;

plot(S1Pupper,ambiguousUpper,'r--o','MarkerSize',5,'MarkerFaceColor','r','LineWidth',2);

f=ambiguousUpper;

end

function f=membershipS1Pupper(x,para,delta)

yS1P=[];

for n=1:x

yS1P=[yS1P;S1PfuncUpper(para(n),delta)];

end

f=yS1P

end

%S1PfuncUpper() is the changed membership function due to the change %in the upper cut off value by an amount of delta. This is derived by %changing the upper cut off value of 1.5 by an amount delta in the %equation (1)

function f=S1PfuncUpper(x,delta)

if x<0.5

f=0;

elseif x<=1.5+delta

f=(x-0.5)/((1.5+delta)-0.5);

elseif x>1.5+delta

f=1;

else

f=NaN;

end

end
